# The combination of *Clostridium butyricum* and *Akkermansia muciniphila* mitigates DSS-induced colitis and attenuates colitis-associated tumorigenesis by modulating gut microbiota and reducing CD8^+^ T cells in mice

**DOI:** 10.1128/msystems.01567-24

**Published:** 2025-01-22

**Authors:** Dengxiong Hua, Qin Yang, Xiaowei Li, Xuexue Zhou, Yingqian Kang, Yan Zhao, Daoyan Wu, Zhengrong Zhang, Boyan Li, Xinxin Wang, Xiaolan Qi, Zhenghong Chen, Guzhen Cui, Wei Hong

**Affiliations:** 1 Key Laboratory of Microbiology and Parasitology of Education Department of Guizhou, Guizhou Medical University 74628 , Guiyang, China; 2 Key Laboratory of Endemic and Ethnic Diseases, Ministry of Education & School/Hospital of Stomatology Guizhou Medical University, Guiyang, Guizhou, China; 3 Collaborative Innovation Center for Prevention and Control of Endemic and Ethnic Regional Diseases Co-constructed by the Province and Ministry & Joint Laboratory of Helicobacter Pylori and Intestinal Microecology of Affiliated Hospital of Guizhou Medical University, Guiyang, Guizhou, China; 4 Guizhou Key Laboratory of Microbio and Infectious Disease Prevention & Control, Guiyang, Guizhou, China; 5 School of Public Health, Guizhou Medical University 74628 , Guiyang, Guizhou, China; 6 Collaborative Innovation Center for Prevention and Control of Endemic and Ethnic Regional Diseases Co-constructed by the Province and Ministry, Guiyang, Guizhou, China; University of California San Diego, La Jolla, California, USA

**Keywords:** probiotic bacteria, *Clostridium butyricum*, *Akkermansia muciniphila*, inflammatory bowel disease, colitis-associated colorectal cancer

## Abstract

The gut microbiota is closely associated with inflammatory bowel disease (IBD) and colorectal cancer (CRC). Probiotics such as *Clostridium butyricum* (CB) or *Akkermansia muciniphila* (AKK) have the potential to treat inflammatory bowel disease (IBD) or colorectal cancer (CRC). However, research on the combined therapeutic effects and immunomodulatory mechanisms of CB and AKK in treating IBD or CRC has never been studied. This study evaluates the potential of co-administration of CB and AKK in treating DSS/AOM-induced IBD and colitis-associated CRC. Our results indicate that compared to mono-administration, the co-administration of CB and AKK not only significantly alleviates symptoms such as weight loss, colon shortening, and increased Disease Activity Index in IBD mice but also regulates the gut microbiota composition and effectively suppresses colonic inflammatory responses. In the colitis-associated CRC mice model, a combination of CB and AKK significantly alleviates weight loss and markedly reduces inflammatory infiltration of macrophages and cytotoxic T lymphocytes (CTLs) in the colon, thereby regulating anti-tumor immunity and inhibiting the occurrence of inflammation-induced CRC. In addition, we found that the combined probiotic therapy of CB and AKK can enhance the sensitivity of colitis-associated CRC mice to the immune checkpoint inhibitor anti-mouse PD-L1 (aPD-L1), significantly improving the anti-tumor efficacy of immunotherapy and the survival rate of colitis-associated CRC mice. Furthermore, fecal microbiota transplantation therapy showed that transplanting feces from CRC mice treated with the co-administration of CB and AKK into other CRC mice alleviated the tumor loads in the colon and significantly extended their survival rate. Our study suggests that the combined use of two probiotics, CB and AKK, can not only alleviate chronic intestinal inflammation but also inhibit the progression to CRC. This may be a natural and relatively safe method to support the gut microbiota and enhance the host’s immunity against cancer.

**IMPORTANCE:**

Our study suggests that the combined administration of CB and AKK probiotics, as opposed to a single probiotic strain, holds considerable promise in preventing the advancement of IBD to CRC. This synergistic effect is attributed to the ability of this probiotic combination to more effectively modulate the gut microbiota, curb inflammatory reactions, bolster the efficacy of immunotherapeutic approaches, and optimize treatment results via fecal microbiota transplantation.

## INTRODUCTION

Probiotics in food can help maintain the balance of the gut microbiota by promoting the growth of beneficial bacteria and inhibiting the reproduction of harmful bacteria, supplementing with probiotics can help alleviate gastrointestinal diseases. Inflammatory bowel disease (IBD), encompassing Crohn’s disease (CD) and ulcerative colitis (UC), is a chronic inflammatory disorder of the colon and small intestine. The etiology and pathogenesis of IBD have been linked to impaired intestinal mucosal barrier function and gut microbiota dysbiosis (1, 2). Conventional clinical interventions for IBD include aminosalicylates, antibiotics, corticosteroids, and immunosuppressants (3). However, these medications often fail to fundamentally address the underlying gut mucosal damage, impaired barrier function, and microbiota dysregulation in IBD patients (4). Moreover, long-term use of these drugs can lead to severe adverse reactions such as nausea, headaches, acne, and nasopharyngitis (4, 5). Notably, IBD patients are at an increased risk of developing colorectal cancer (CRC) (6). Current preventive strategies for CRC include lifestyle modifications, screening high-risk individuals, surgical polyp removal, and early aspirin intervention. Treatment for CRC primarily involves surgical resection combined with chemotherapy, radiotherapy, or targeted therapies (7). However, the emergence of chemotherapy resistance and the limited efficacy of immunotherapy highlight the need for novel therapeutic approaches (8).

Accumulating evidence suggests that gut microbiota dysbiosis plays a crucial role in the pathogenesis of various gastrointestinal (GI) diseases, including celiac disease (9), and CRC (10). Gut microbiota perturbations lead to a reduction of beneficial bacteria and overgrowth of certain pathogens. Oral administration of probiotics or fecal microbiota transplantation (FMT) can restore gut microbiota balance, alleviate dysbiosis, and mitigate disease progression (11). Emerging probiotic strains, such as *Clostridium butyricum* (CB) (12–14), *Akkermansia muciniphila* (AKK) (15, 16), *Roseburia intestinalis* (Ri) (17), and *Lactobacillus reuteri* (Lr) (18), have demonstrated promising therapeutic potential in animal models, including regulating host immune responses, alleviating intestinal inflammation, suppressing tumor cell proliferation, inducing apoptosis, and enhancing the sensitivity to immunotherapy or chemotherapy.

CB and its metabolic products have multiple potential applications in food processing, including enhancing food flavor, preservation, antioxidant properties, and vitamin fortification. Furthermore, CB, *Faecalibacterium,* and *Roseburia* spp. are genera capable of producing short-chain fatty acids (SCFAs), among which butyrate can provide energy for epithelial cells, which is beneficial for maintaining intestinal health (19), which maintains colonocyte homeostasis and intestinal barrier integrity. Studies have shown that in mice with IBD and CRC, CB not only effectively modulates the gut microbiota composition and reduces pathogenic bacteria in the intestine but also diminishes the production of pro-inflammatory cytokines like interleukin-1β (IL-1β), interleukin-6 (IL-6), and tumor necrosis factor-α (TNF-α) (20). It significantly downregulates signaling pathways such as myeloid differentiation primary response protein 88 (MYD88), nuclear factor kappa-B (NF-κB), and Wnt/β-catenin (WNT) (21, 22), thereby preventing the formation of inflammation-associated CRC. AKK, a star strain, is a Gram-negative anaerobe and the first and only representative member of the *Verrucomicrobia phylum* found in the human gut. Numerous studies have found that AKK can benefit systemic metabolism, suppress intestinal inflammation, maintain intestinal barrier integrity, and anti-tumor immunity (15, 23, 24). For instance, the abundance of AKK decreases significantly in metabolic diseases such as diabetes and obesity. In obese human volunteers, pasteurized AKK supplements can improve host metabolic dysfunction and intestinal barrier integrity and reduce serum cholesterol, triglyceride, and lipopolysaccharide levels (25). Moreover, AKK can activate the adaptive immune response by regulating the infiltration of macrophages and CD8^+^ T cells in the CRC tumor site, thus maintaining the host’s immune homeostasis (24).

**Fig 1:**
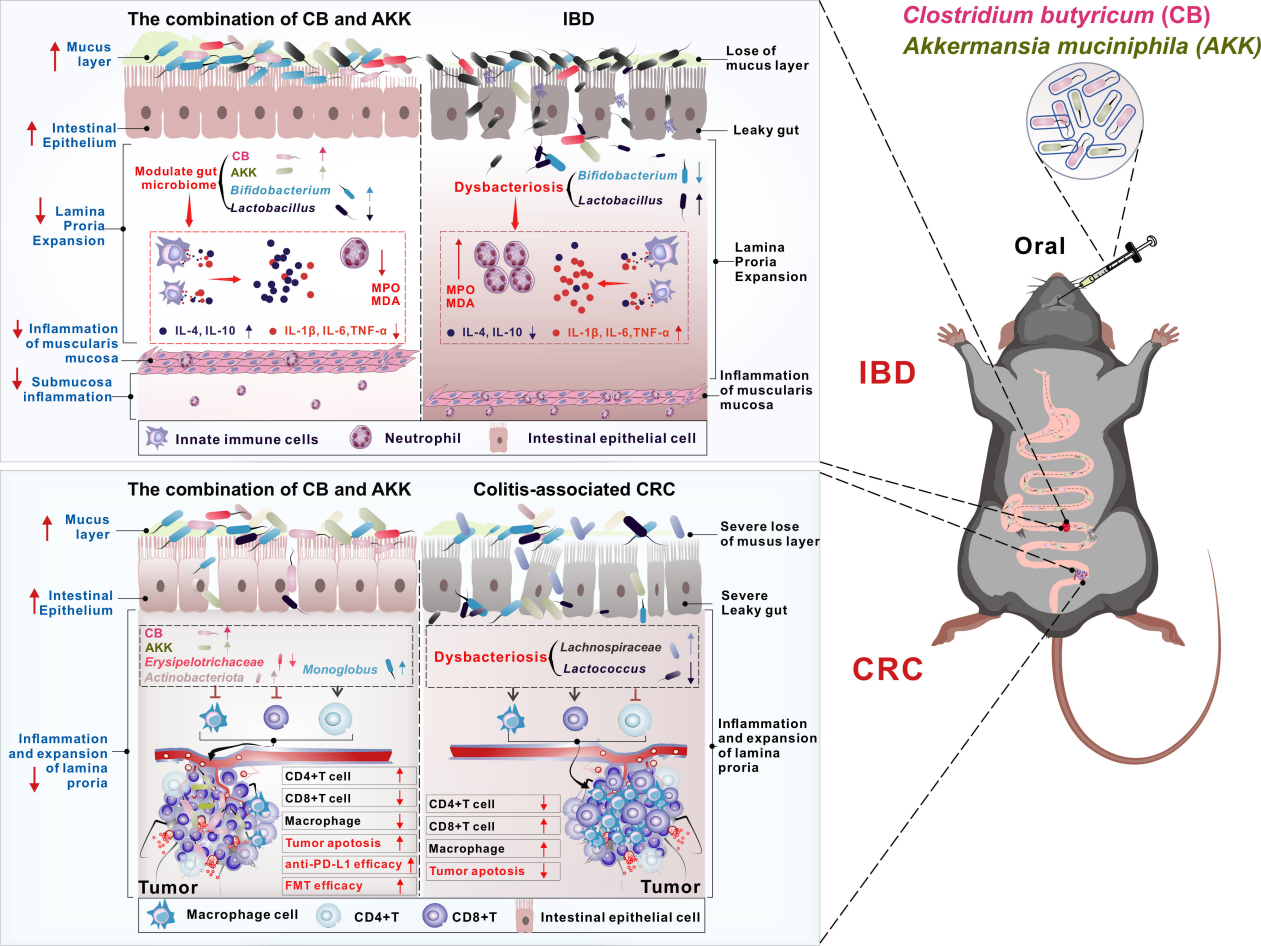
In IBD mouse models, the combination of CB and AKK can significantly improve intestinal mucosal barrier damage, alleviate intestinal leakage, regulate the imbalance of *Bifidobacterium* and *Lactobacillus* in the gut, and reduce the levels of IL-1β, IL-6, TNF-α, myeloperoxidase (MPO), and malondialdehyde (MDA) in the intestine, further alleviating oxidative stress damage and inflammatory responses induced by DSS. In colitis-associated CRC mouse models, oral administration of CB and AKK not only alleviates damage to the intestinal barrier and reduces the levels of inflammation-associated CD8^+^ T cells and macrophages in the gut, inhibits tumor growth, and promotes tumor cell apoptosis; it also increases the sensitivity of colitis-associated CRC mice to the immune checkpoint inhibitor drug anti-PD-L1 (aPD-L1), and fecal microbiota transplantation (FMT) based on CB and AKK can extend the survival rate of colitis-associated CRC mice. Taken together, the combined use of the two probiotics, CB and AKK, has strong potential in treating IBD or colitis-associated tumorigenesis.

Although both CB and AKK, as gut probiotics, have been shown to provide beneficial effects in maintaining the intestinal homeostasis of the host, and studies have demonstrated their protective roles against IBD and CRC in mice, however, the research on therapeutic effects and mechanisms of these two probiotics used in combination for the treatment of IBD or colitis-associated CRC (the disease, induced by long-term DSS administration, is a consequence of untreated IBD leading to cancerous transformation) have never been reported. Particularly, there is a lack of information on whether the combination of CB and AKK can better regulate the gut microbiota and enhance the host anti-tumor immune response to colitis-associated CRC. Furthermore, the potential of combined probiotic therapy of CB and AKK to enhance the sensitivity and efficacy of the host to immunotherapeutic drugs such as anti-programmed cell death ligand 1 monoclonal antibody (aPD-L1) requires further investigation. Therefore, to explore these questions, we first observed the effects and immune regulatory mechanisms of the combination of CB and AKK on two different intestinal disease models of IBD and colitis-associated CRC; second, in colitis-associated CRC mice, we also investigated the changes in the sensitivity to immune therapeutic drug aPD-L1 and immune regulatory function of tumor mice after combination of CB and AKK; third, we also preliminarily explored the intervention effect of FMT therapy derived from CB and AKK treatment on colitis-associated CRC mouse (as illustrated in Fig. 1).

## RESULTS

### The combination of CB and AKK alleviates DSS-induced colitis mice

**Fig 2:**
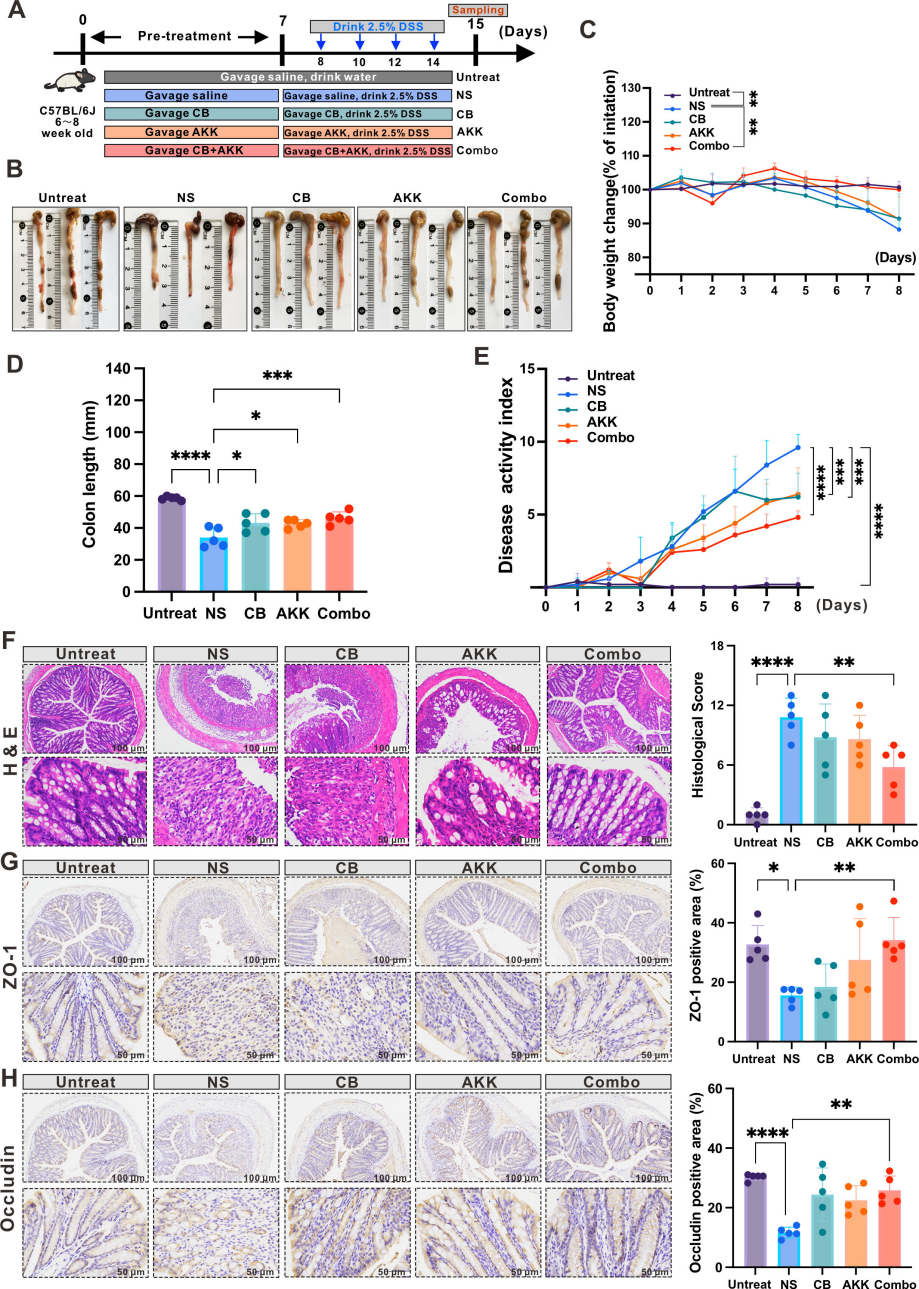
The combination of CB and AKK mitigates DSS-induced colon inflammation and enhances the integrity of the colonic barrier in mice. (**A**) A schematic diagram of the DSS-induced colitis model in mice treated with saline (NS group), CB (CB group), AKK (AKK group), and a combination of CB, AKK (Combo group), and without DSS (Untreated group), respectively. (**B**) Representative photographs of the colon and corresponding measurements of colon length (*n* = 5 per group). (**C**) The daily weight change rate for mice was determined by dividing the weight on each day by the initial weight at the start of the experiment, with data expressed as mean ± SEM (*n* = 5 per group). Statistical significance among groups on the final day of the model (day 8) was assessed using one-way ANOVA, followed by Tukey’s test for multiple comparisons. Significance levels relative to the normal saline (NS) group are indicated by *P ≤ 0.05, **P ≤ 0.01, ***P ≤ 0.001, ****P ≤ 0.0001. (**D**) Average colon lengths for each treatment group. (**E**) Disease activity index (DAI) scores. (**F**) Histological evaluation with hematoxylin and eosin (H&E) staining and scoring of colon tissue. (**G**) Immunohistochemical analysis of the tight junction proteins ZO-1 and (**H**) occludin in colonic tissue sections from mice, with quantification of positive staining areas. Scale bars represent 50 µm and 100 µm, respectively. Statistical significance compared to the NS group is denoted by *P ≤ 0.05, **P ≤ 0.01, ***P ≤ 0.001, ****P ≤ 0.0001.

To assess the effects of the combination of CB and AKK on DSS-induced colitis in mice, we pre-treated mice with saline (NS group), CB (CB group), AKK (AKK group), and CB + AKK (Combo group) for 1 week before inducing colitis in mice using DSS; gavage saline only and without treated with DSS (Untreated group) (four times, every 2 days, Fig. 2A). After the treatment, we observed the changes in body weight, occurrence of diarrhea, fecal occult blood, colon length, and spleen weight in each group of mice. As shown in Fig. 2B through E, compared with the NS group, both the single bacteria group (CB group or AKK group) and Combo group can significantly alleviate the DAI score increase and colon shortening of DSS mice. By contrast, the Combo group can further alleviate the weight loss of mice (Fig. 2C). These results suggest that the combination of CB and AKK can significantly alleviate the colon shortening and colitis symptoms induced by DSS.

To further explore whether the combination of CB and AKK can alleviate the damage of colon mucosal barrier function induced by DSS, we first conducted a histopathological evaluation of the colon tissue in each group of mice. Hematoxylin and eosin (H&E) results showed (Fig. 2F) that compared with the Untreated group, colon tissue in the NS group showed a large number of crypt loss, epithelial surface damage, decreased mucin secretion, and infiltration of immunocytes. However, the Combo treatment significantly reduced the occurrence of these conditions. Transmembrane proteins (such as occludin and claudins) and tight junction proteins (such as ZO-1) can be used to evaluate the integrity of the intestinal mucosal barrier. Immunohistochemistry (IHC) results showed that, compared with the Untreated group, the expression levels of colon barrier functional proteins ZO-1 and occludin were decreased in the NS group. By contrast, the expression level of colonic barrier proteins ZO-1 and occludin in DSS mice increased significantly in the Combo group than in the single bacteria group (CB or AKK group; Fig. 2G and H). These results indicate that the combination of CB and AKK can partially restore the damage to DSS-induced colon barrier function.

### The combination of CB and AKK reduces inflammation in DSS-induced colitis mice

**Fig 3:**
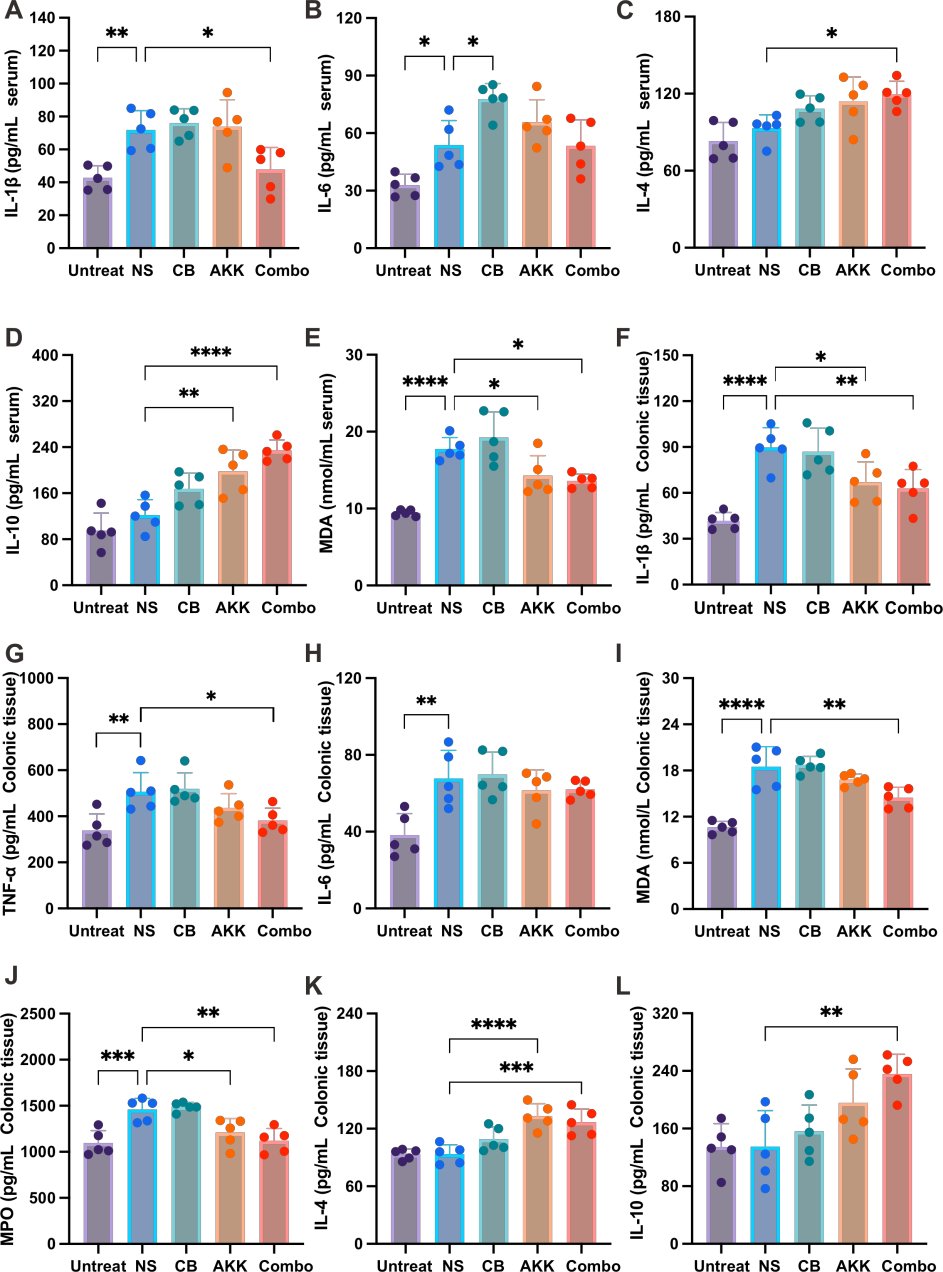
The combination of CB and AKK attenuates the inflammatory response in DSS-induced colitis in mice. Concentrations of three representative pro-inflammatory cytokines, IL-1β (**A**), IL-6 (**B**), and MDA (**E**), as well as two representative anti-inflammatory cytokines, IL-4 (**C**) and IL-10 (**D**), in serum. The concentrations of IL-1β (**F**), TNF-α (**G**), IL-6 (**H**), MDA (**I**), MPO (**J**), IL-4 (**K**), and IL-10 (**L**) in the colon tissue. Data are presented as mean ± SD. Statistical significance was determined using one-way ANOVA, followed by Tukey’s *post hoc* test. *P ≤ 0.05, **P ≤ 0.01, ***P ≤ 0.001, ****P ≤ 0.0001.

Inflammatory cytokine levels in colitis are closely correlated with the severity of inflammation. We used ELISA kits to evaluate the levels of pro-inflammatory and anti-inflammatory factors in the serum and colon tissues of mice in each group. The results showed that the levels of pro-inflammatory factors IL-1β (Fig. 3A) and IL-6 (Fig. 3B) were significantly increased in the serum of the NS group compared with the Untreated group. However, the combination of CB and AKK significantly reduced the increase in IL-1β level (Fig. 3A) and significantly increased the expression of serum anti-inflammatory factors IL-4 (Fig. 3C) and IL-10 (Fig. 3D). Furthermore, we indirectly evaluated the degree of tissue peroxidation damage of mice in each group by detecting the level of serum MDA. Compared with the NS group, serum MDA levels in the AKK and Combo groups were significantly decreased (Fig. 3E), suggesting that CB and AKK treatment improved the antioxidant activity of DSS-induced mice.

In addition, we further evaluated the levels of pro-inflammatory factors IL-1β, IL-6, TNF-α, LPS, MPO, and MDA, and anti-inflammatory factors IL-4 and IL-10 in the colonic tissues of mice. The results showed that compared with the Untreated group, the NS group showed significantly increased expression of pro-inflammatory factors IL-1β (Fig. 3F), TNF-α (Fig. 3G), IL-6 (Fig. 3H), MDA (Fig. 3I), and MPO (Fig. 3J) after DSS treatment. The combination of CB and AKK significantly reduced the expression of pro-inflammatory factors IL-1β (Fig. 3F), TNF-α (Fig. 3G), MDA (Fig. 3I), and MPO (Fig. 3J) in the colonic tissues and also increased the expression of anti-inflammatory factors IL-4 (Fig. 3K) and IL-10 (Fig. 3L), which is in line with inflammatory cytokine levels in the serum. In conclusion, the above results suggest that the combination of CB and AKK can inhibit inflammation in DSS-induced mice.

### The combination of CB and AKK regulates gut microbiota dysbiosis in DSS-induced IBD model

**Fig 4:**
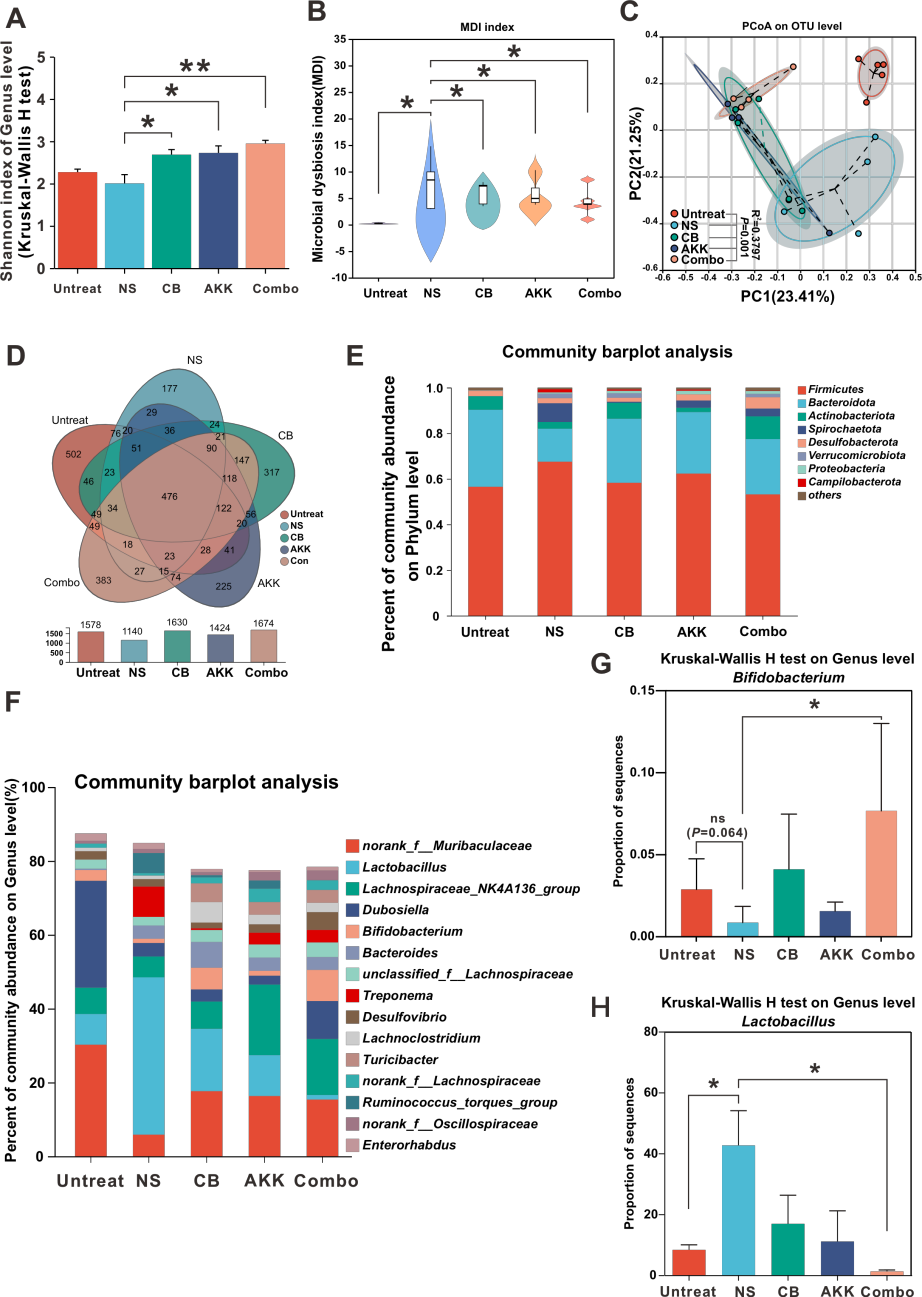
The combination of CB and AKK regulates gut microbiota dysbiosis in the DSS-induced IBD model. (**A**) Assessment of the α-diversity of the gut microbiota using the Shannon index (*n* = 5). (**B**) Evaluation of the dysbiosis degree of the gut microbiota in four groups of DSS-induced IBC mice models using the dysbiosis index (*n* = 5 per group), with a two-tailed Wilcoxon rank-sum test for inter-group MDI comparison at the OUT level and FDR correction applied to *P*-values, *P ≤ 0.05, **P ≤ 0.01, ***P ≤ 0.001. (**C**) Differential assessment of the relative abundance of fecal microbiota among five groups in principal coordinate analysis (PcoA) using PERMANOVA, with sample distances calculated using the Bray-Curtis method. (**D**) Venn diagram analysis of the composition of gut microbiota. (**E**) Analysis of the phylum level and (**F**) genus level of gut microbiota in the feces of mice from each group. (**G**) Comparative analysis of the relative abundance of *Bifidobacterium* and (**H**) *Lactobacillus* at the genus level in the gut microbiota of mice from each group.

To study the effects of the combination of CB and AKK on the gut microbiota in the DSS-induced IBD mice model, we performed high-throughput sequencing of 16S rRNA genes in the cecal contents of each group of mice to analyze the composition of the gut microbiota in each group of mice. The results of alpha diversity evaluation showed that at the Genus level, the Shannon index significantly increased in the CB, AKK, and Combo groups compared with the NS group (Fig. 4A). Second, the results of the microbial dysbiosis index (MDI) analysis also showed that the MDI of the NS group was significantly higher than the CB, AKK, Combo, and Untreated groups (Fig. 4B). These results suggest that the administration of CB, AKK, or Combo can effectively increase the diversity of the gut microbiota in the IBD mice model, thereby reducing the degree of gut microbiota dysbiosis in DSS mice.

Principal coordinate analysis (PCoA) was performed using Bray-Curtis distance measurement (Fig. 4C). The results showed that the Combo group mice formed a distinct cluster from other groups, while no significant difference was found between the CB and AKK groups. Furthermore, the cluster of the Untreated group was closer to the Con-treated cluster, indicating that CB and AKK combination had partially restored the gut microbiota of IBD mice, and the effect of Combo treatment on recovering gut microbiota was better than that of monophonic CB or AKK treatment groups. The Venn diagram results of species composition analysis showed the following (Fig. 4D): (i) at the genus level, there were 476 common genera between groups; (ii) the number of unique genera in the Untreated group, NS group, CB group, AKK group, and Combo group was as follows: Untreated group had the most unique genera, with a total of 502; NS group had the fewest unique genera, with only 177; CB group had 317 unique genera, AKK group had 225 unique genera, and Combo group had 383 unique genera, ranking best in the intervention groups. The results suggested that the intestinal microbial community of mice in the NS group was damaged compared with that in the Untreated group. However, after Combo intervention, the intestinal microbial community structure was restored to a considerable extent.

To further compare the microbial composition between different groups, we identified the most abundant bacterial populations at the phylum and genus levels (Fig. 4F and E). At the phylum level, the gut microbiota composition analysis, as shown in Fig. 4E, compared with the Untreated group, the relative abundance of the main branches representing the intestinal microbiota (*Bacteroidota, Firmicutes, Proteobacteria,* and *Actinobacteria*) in the NS group changed greatly, among which the percentage of *Bacteroidota* and *Actinobacteria* decreased significantly, while that of *Firmicutes* increased significantly. However, the intervention of CB, AKK, or CB + AKK in IBD mice partially reversed the changes in *Bacteroidota*, *Actinobacteria,* and *Firmicutes* at the phylum level. Furthermore, gut microbiota composition in the Combo group was the closest to that of the Untreated group.

**Fig 5:**
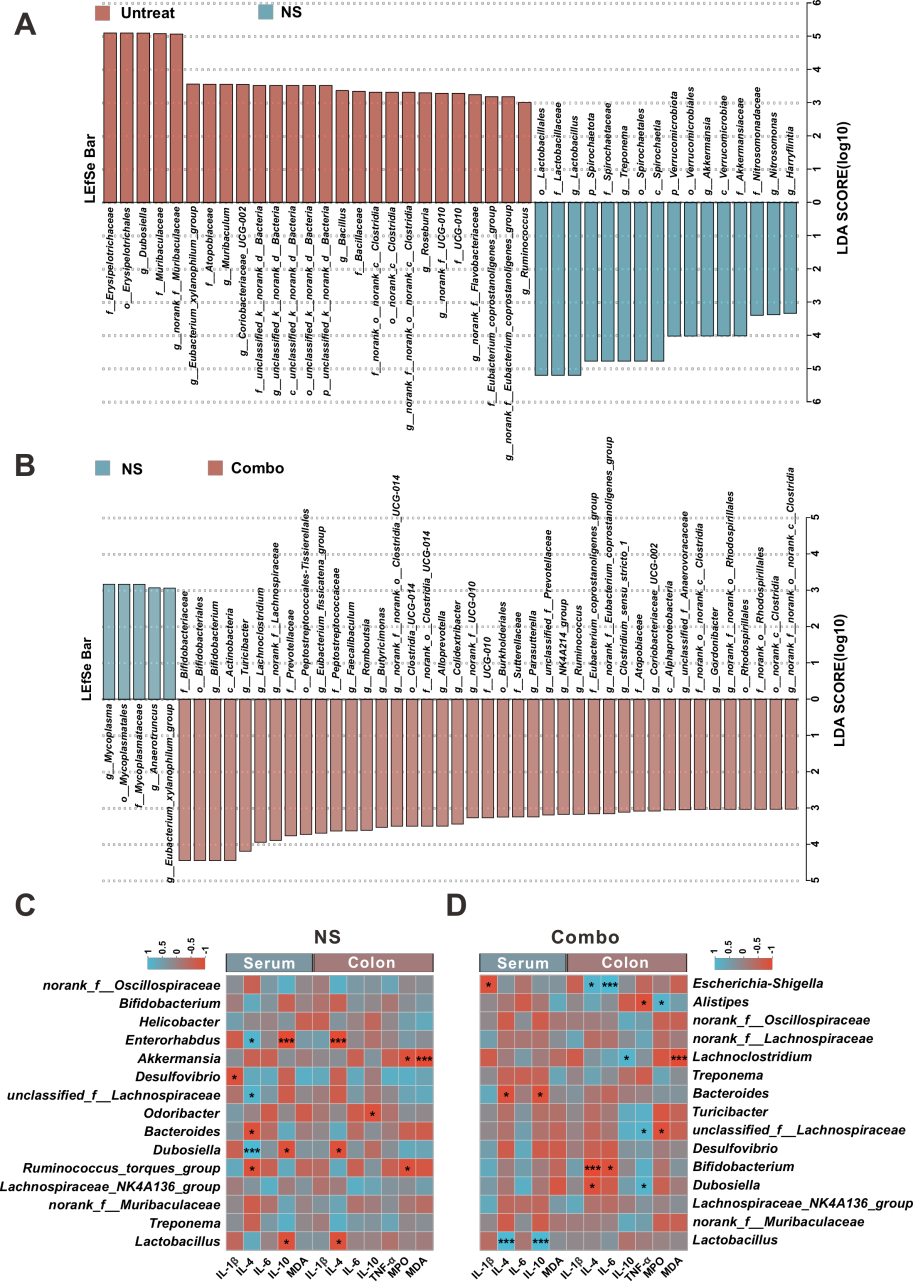
Analysis of the gut microbiota in mice with DSS-induced IBD model using LEfSe and Spearman correlation analysis. (**A**) Analysis of the microbial taxa difference between the Untreated group and the NS group using LEfSe (LDA > 3). (**B**) Analysis of the microbial taxa difference between the NS group and the Combo group using LEfSe (LDA > 3). (**C**) Heatmap of Spearman correlation between intestinal microbiota and colitis-related phenotype in NS group and Combo group (**D**).

Compared with the NS group, the abundance of *norank_f_Muribaculaceae* was significantly increased after treatment with CB, AKK, or Combo at the genus level. Second, compared with the NS group, we observed a significant increase in *Bifidobacterium* abundance in the Combo group (Fig. 4G). Interestingly, our results surprisingly showed that the abundance of *Lactobacilli* was significantly higher in the NS group than in the Untreated group. By contrast, in the Combo group, the abundance of *Lactobacilli* was significantly lower than that in the NS group (Fig. 4H). However, *Lactobacilli* is generally considered to be a beneficial probiotic that is beneficial to the treatment of IBD (Fig. 4H). In addition, we use LEfSe analysis to determine the differences in microbial communities between different groups. By comparing the Untreated group and NS group, we found that when LDA > 3, the Untreated group was significantly enriched in the *norank_f_Muribaculaceae*, which is associated with anti-inflammatory effects (Fig. 5A). By comparing the NS group and Combo group, the LDA score of *Bifidobacterium* in the Combo group was significantly higher than that in the NS group (Fig. 5B), which is consistent with the abundant bacterial populations analysis at the genus level (Fig. 4F and G).

### The combination of CB and AKK can reduce colonic inflammation in IBD mice by regulating the proportion of *Bifidobacterium* and *Lactobacillus* in the gut

We further analyzed the impact of the gut microbiota on anti-inflammatory and inflammatory factors in the NS and Combo groups. We used Spearman correlation analysis to predict the correlation between the gut microbiota of the two groups of mice and the corresponding pro- and anti-inflammatory factors in the serum and colonic tissue (Fig. 5C and D). The results of the NS group analysis showed that AKK was negatively correlated with MPO (inflammatory) and MDA (inflammatory) in the colon (Fig. 5C). *Lactobacilli* was negatively correlated with IL-10 (anti-oxidative) and IL-4 (anti-inflammation) in the serum and colonic tissue in the NS group (Fig. 5C), suggesting that this genus may promote colon inflammation. In addition, *Enterorhabdus* and *Dubosiella* may have the same role as *Lactobacilli* in promoting inflammation in the NS group (Fig. 5C), which also showed a negative correlation with IL-10 (anti-inflammation) and IL-4 (anti-inflammation) in the serum and colonic tissue.

Meanwhile, in the Combo group (Fig. 5C), the *Lactobacilli* had a significant positive correlation with IL-4 and IL-10, which is reversed to the result observed in the NS group. *Bifidobacterium* was negatively correlated with IL-6 and IL-4 in the colon (Fig. 5D). These results suggest that administration of CB + AKK could reverse the pro-inflammation effects of *Lactobacilli* and promote the anti-inflammation effect of *Bifidobacterium*, alleviating inflammation in IBD.

### The combination of Combo with aPD-L1 alleviates the clinical symptoms of colitis-associated CRC in a mouse model

**Fig 6:**
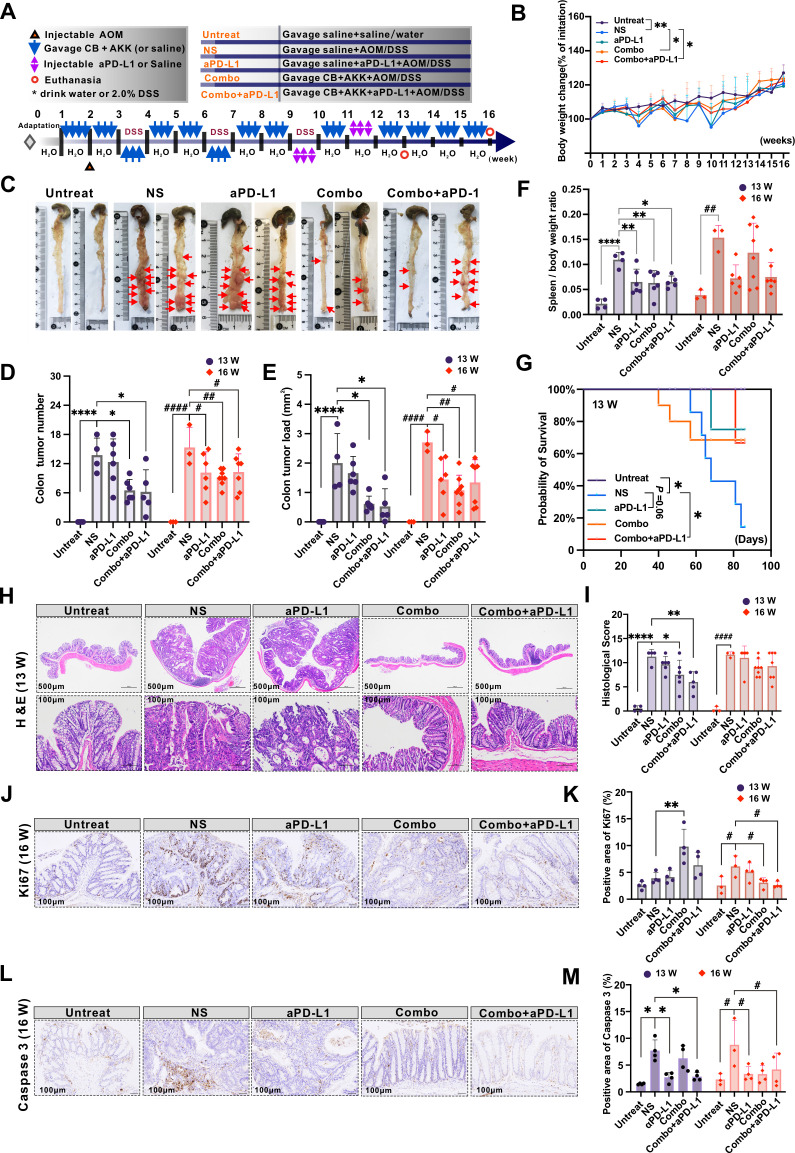
The combination of Combo with aPD-L1 alleviates the clinical symptoms of colitis-associated CRC in the mouse model. (**A**) Schematic representation of the colitis-associated CRC mouse model, with treatments administered via oral gavage of saline (Untreated group), CB + AKK (Combo group), NS group intraperitoneal injection of aPD-L1 (aPD-L1 group), and Combo group intraperitoneal injection of aPD-L1 (Combo + aPD-L1 group). The blue arrows indicate that the mice were orally gavaged with bacteria. (**B**) Body weight change rates of mice in each group (*n* = 5). (**C**) Representative colon images of colitis-associated CRC mice at 13 and 16 weeks, the red arrows indicate tumors that develop in the mice’s colon. (**D**) Tumor count and (**E**) tumor area calculation in the colon of colitis-associated CRC mice. (**F**) Spleen-to-body weight ratios in mice at 13 and 16 weeks. (**G**) Survival curve analysis of mice in each group at 13 weeks. (**H**) H&E staining of mouse colon at 13 weeks. (**I**) Histopathological scores of mouse colon at 13 and 16 weeks. (**J**) Immunohistochemical staining of Ki67 in mouse colon at 16 weeks. (**K**) Calculation of the positive staining area of Ki67 immunohistochemistry in mouse colon at 13 and 16 weeks. (**L**) Immunohistochemical staining of Caspase 3 in mouse colon at 16 weeks. (**M**) Calculation of the positive staining area of Caspase 3 immunohistochemistry in mouse colon at 13 and 16 weeks. Data are presented as mean ± SD and were analyzed using one-way ANOVA with Tukey’s multiple comparison test. Compared with the NS group, *P < 0.05, **P < 0.01, ***P < 0.001, ****P < 0.0001 or #*P* < 0.05, ##*P* < 0.01, ###*P* < 0.001, ####*P* < 0.0001.

The above results indicate that the Combo group is the most effective in alleviating IBD symptoms, inhibiting inflammatory factors, and restoring the gut microbiota composition. Therefore, in the following work, we focus on the therapeutic effect of the Combo group and immune checkpoint therapy (ICT) on colitis-associated CRC. Furthermore, in the colitis-associated CRC mouse model, the therapeutic effects of Combo and Combo + aPD-L1 intervention were evaluated at two different time points: 13 weeks (13W) and 16 weeks (16W) post-tumor implantation (Fig. 6A).

Compared with the Untreated group, the NS group showed significant weight loss (Fig. 6B), many tumors in the colon (Fig. 6C through E), a significant increase in spleen/weight ratio (Fig. 6F), and a high mortality rate in the modeling process (Fig. 6G). After the intervention by Combo or Combo + aPD-L1, compared with the NS group, it was observed that the clinical manifestations of colitis-associated CRC mice in the above two groups were significantly improved at 13 W and 16W, including weight loss (Fig. 6B), splenomegaly (Fig. 6F), and significantly reduced the formation of tumors in the colon (Fig. 6E though E). In addition, survival analysis of colitis-associated CRC mice at 13W duration showed a significant reduction in survival of colitis-associated CRC mice in the NS group compared to the Untreated group. However, after Combo + aPD-L1 intervention, the survival rate of colitis-associated CRC mice was significantly improved (Fig. 6G).

Histological results further showed that at 13W and 16W duration, compared with the Untreated group, the colon tissue of NS group mice had severe mucosal damage, large areas or almost complete loss of crypts, abnormal proliferation of epithelial cells, and accompanied by a large number of inflammatory cell infiltration (Fig. 6H and I). However, compared with the NS group, the Combo or Combo + aPD-L1 group significantly alleviated the pathological changes such as colonic mucosal injury, large area or absence of crypts, abnormal proliferation of epithelial cells, and inflammatory cell infiltration in the colitis-associated CRC mouse group at 13 W (Fig. 6H and I). In the Combo group, the level of Ki67, a cell proliferation marker, was significantly higher than in NS at 13 W (Fig. 6K). However, its expression in the colon was reduced considerably after Combo or Combo + aPD-L1 intervention at 16 W. In addition, at 16 W, Combo or Combo + aPD-L1 treatment significantly reduced the expression of Ki67 in the colon of colitis-associated CRC mice (Fig. 6J and K).

Similarly, we observed a significant downregulation of Caspase 3 expression in the colon of colitis-associated CRC mice at 13 W and 16 W following aPD-L1 or Combo + aPD-L1 intervention (Fig. 6L and M). These results suggest that Combo or Combo + aPD-L1 can inhibit the proliferation of tumor cells and improve the clinical symptoms of colitis-associated CRC mice compared with the NS group. The Combo + aPD-L1 group showed better cancer inhibition effects.

### The combination of Combo with aPD-L1 can regulate the immune response of colitis-associated CRC mice model

**Fig 7:**
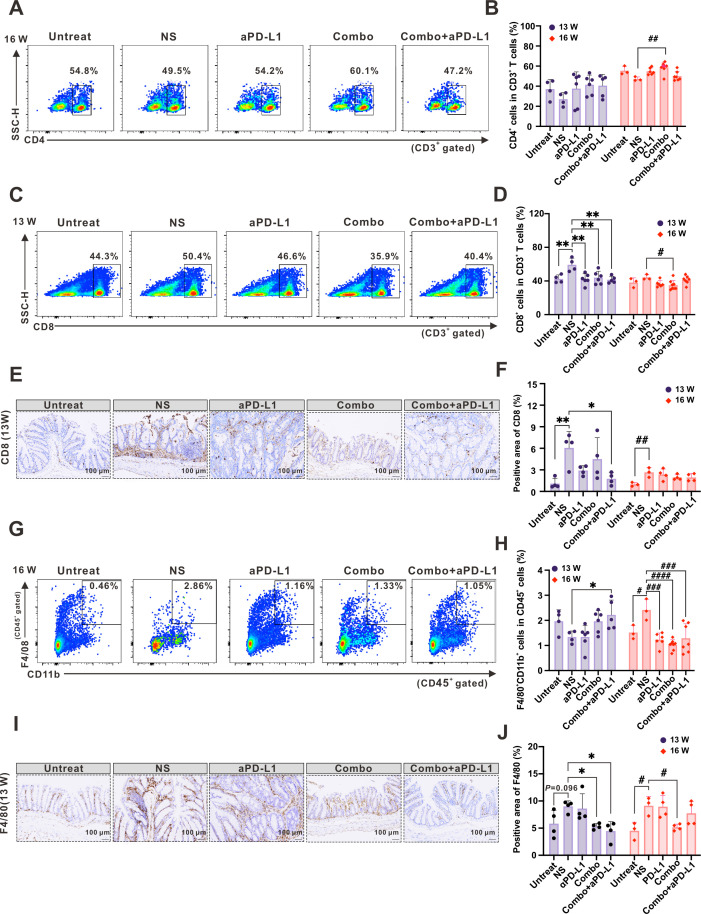
The combination of Combo with aPD-L1 can regulate the immune response of the colitis-associated CRC mice model. (A–D) Analysis and quantification of CD4^+^ T and CD8^+^ T cells in the mice spleen using FCM. (E) IHC of CD8a in mice colon and quantitative analysis of its positive rate by Image J software (F), scale bars: 100 µm. (G and H) Analysis and quantification of macrophage cells in the mice spleen using FCM. (I) IHC of F4/80 in mice colon and quantitative analysis of its positive rate by Image J software (J), scale bars: 100 µm. Data are presented as the mean ± SD and were analyzed by ordinary one-way ANOVA with Tukey’s multiple comparisons. *P < 0.05, **P < 0.01, ***P < 0.001 or #*P* < 0.05, ##*P* < 0.01, ###*P* < 0.001, compared with NS group.

In the context of anti-tumor immunity, the immune system plays an important role. We used flow cytometry (FCM) to detect the levels of CD4^+^ T, CD8^+^ T, macrophages, and M1 (M1-type macrophages, M1) polarization in the spleens of mice in each group. At 16W, the percentage of CD4^+^ T cells in the spleens of colitis-associated CRC mice in the Combo group was significantly higher than in the NS group (Fig. 7A and B). To further evaluate the regulatory T cells (Treg) status in mice in each group, we used FCM to detect the percentage of CD4^+^CD25^+^ T cells in the spleens of mice in each group. The results showed no significant difference in the percentage of Treg cells between groups (Fig. S1). In addition, at 13W, the percentage of CD8^+^ T cells in the spleens of NS mice was significantly higher compared to the other groups (aPD-L1, Combo, and Combo + aPD-L1, Fig. 7C and D). In addition, the percentage of CD8^+^ T in the spleen of the NS group was also significantly higher than that in the Combo group at 16W (Fig. 7C). The results of the IHC analysis conducted on the mice of 13W and 16W also revealed that the percentage of CD8^+^ T cells in the NS group was significantly higher than that in the Untreated group (Fig. 7E and F). Moreover, the administration of Combo + aPD-L1 led to a significant reduction in the percentage of CD8^+^ T cells in the colon of colitis-associated CRC mice.

Macrophages are the main immune cells that mediate immunosuppression in the tumor microenvironment and can suppress anti-tumor immune responses. Studies have shown that in inflammation, CD8^+^ T cells can recruit macrophages, thereby promoting inflammation. Thus, we measured the levels of macrophages and their M1 polarization in the spleen. The results showed that although we did not observe significant differences in the percentage of macrophages with M1 polarization markers (CD11b^+^F4/80^+^CD86^+^) in the spleens of mice at 13 and 16 weeks (Fig. S1B), the percentage of macrophages in the NS group was significantly higher than that in the Untreated group at 16W (Fig. 7G and H). However, the percentage of macrophages in the spleens of mice was significantly reduced after treatment with aPD-L1, Combo, or Combo + aPD-L1 (Fig. 7G and H). Colon macrophage infiltration was significantly reduced with Combo or Combo + aPD-L1 intervention in both 13W and 16W, as confirmed by IHC (Fig. 7I and J). These findings indicate that Combo or Combo + aPD-L1 may elicit anti-inflammatory and anticancer effects in colitis-associated *in situ* CRC mice by decreasing systemic or local inflammatory infiltration of macrophages and CD8^+^T cells.

### The effect of administration of Combo or Combo+aPD-L1 on gut microbiota dysbiosis in the colitis-associated CRC mice

**Fig 8:**
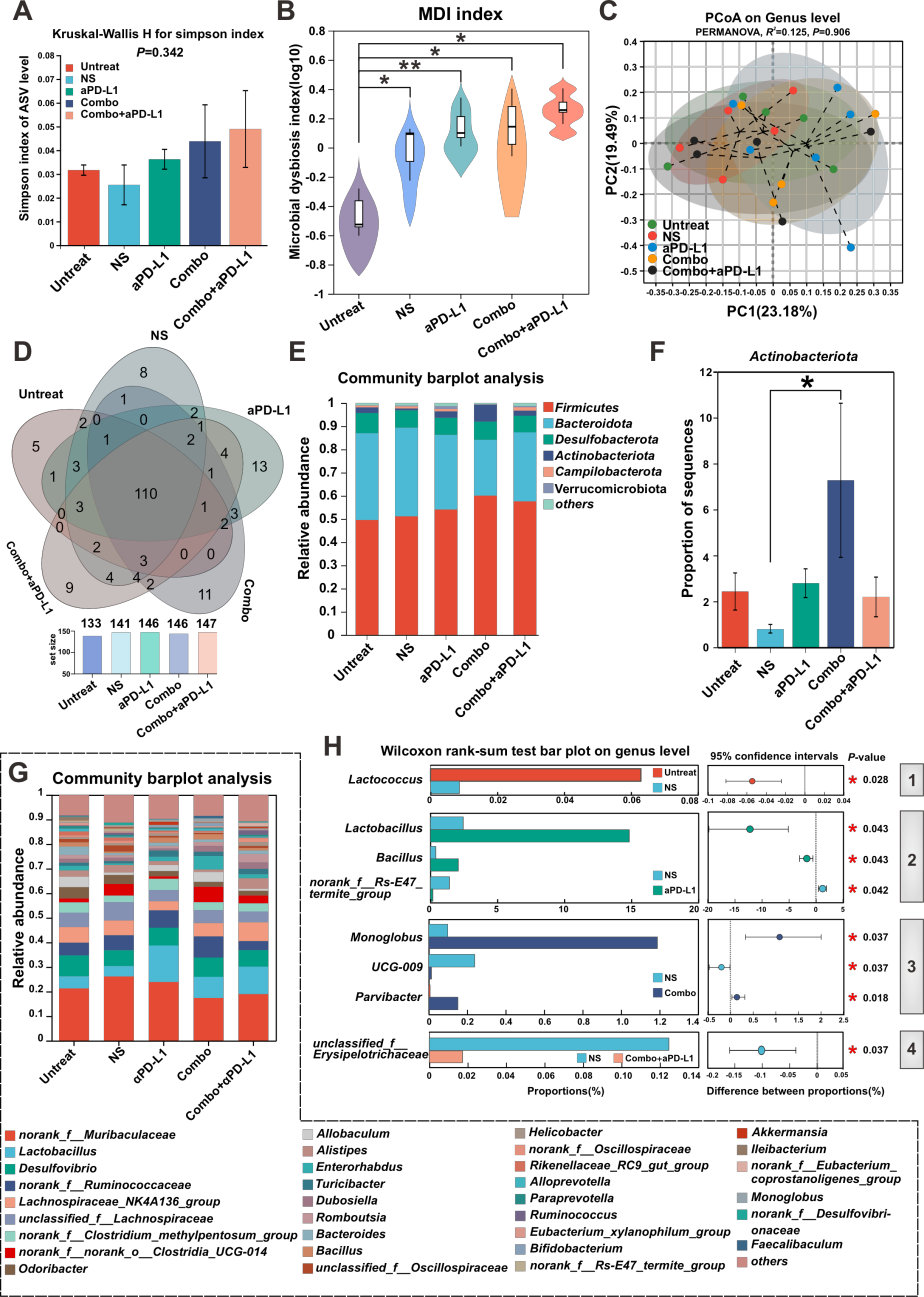
Analysis of gut microbiota in colitis-associated CRC mice model which interventions with Combo, or Combo + aPD-L1. (**A**) Evaluation of the α-diversity of gut community composition using the Simpson index. (**B**) Assessment of gut microbiota dysbiosis in four groups of colitis-associated CRC mice using the MDI, with a two-tailed Wilcoxon rank sum test for inter-group MDI differences at the OUT level, and correction of *P*-values using the FDR method, *P ≤ 0.05, **P ≤ 0.01, ***P ≤ 0.001. (**C**) Differential evaluation of the relative abundance of fecal microbiota among the five groups in PCoA using PERMANOVA at the OUT level, and calculation of inter-sample distances using the Bray-Curtis method. (**D**) Species Venn diagram analysis of gut microbial composition. (**E**) Differences in the relative abundance within each group of mice at the genus level. (**F**) Comparative analysis of the relative abundance of *Actinobacteria* at the phylum level for each group of mice. (**G**) Analysis of the fecal microbiota at the genus level for each group of mice. (**H**) identification of species with significant differences in relative abundance when comparing the NS group with other groups.

To investigate the impact of Combo and Combo + aPD-L1 on the gut microbiota in the colitis-associated CRC mice model, we conducted high-throughput sequencing of the 16S rRNA gene in the cecal contents of 13 W mice of Untreated, NS, aPD-L1, Combo and Combo + aPD-L1 groups. At the amplicon sequence variant (ASV) level, intergroup comparisons revealed no statistically significant differences in α-diversity, such as the Simpson index (Fig. 8A), Shannon index (Fig. S2A), observed species (Sobs) index (Fig. S2B), and Chao index (Fig. S2C). The MDI serves as an indicator of gut microbiota disorder. The MDI significantly increased in the NS group, aPD-L1 group, Combo group, and Combo + aPD-L1 group compared to the Untreated group (Fig. 8B), suggesting varying degrees of gut microbiota disruption in the colitis-associated CRC mice. The PCoA results (Fig. 8C) demonstrated no distinct clustering separation among the treatment groups. In addition, a Venn diagram analysis of species composition among the groups revealed the following (Fig. 8D): (i) at the genus level, 110 shared genera were identified across all groups, and (ii) the numbers of unique genera for the Untreated group, NS group, aPD-L1 group, Combo group, and Combo + aPD-L1 group were 5, 8, 13, 11, and 9, respectively, indicating no significant differences in species composition among the treatment groups.

At the phylum level (Fig. 8E), our further observations indicated no significant changes in the abundance of *Firmicutes*, *Bacteroidota*, and *Desulfobacterota* in the gut of mice from each experimental group compared to the Untreated group. However, in comparison to the NS group, the intervention with Combo in colitis-associated CRC mice significantly increased the abundance of *Actinobacteria* in the gut of these mice (Fig. 8F). *Actinobacteria* includes various genera with potential probiotic effects, such as *Bifidobacterium*, *Actinomyces*, and *Mycobacterium*. Nevertheless, in analyzing genus-level species composition among the groups, we did not observe any significant differences in the abundance of the genera mentioned above, including *Bifidobacterium* (Fig. 8G). Furthermore, when comparing the single-species composition at the genus level among the NS group and the other groups, the following findings were noted (Fig. 8H): (i) compared to the Untreated group, the abundance of the beneficial genus *Lactococcus* was significantly reduced in the gut of NS group mice (Fig. 8H1); (ii) compared with the NS group, the treatment with aPD-L1 (aPD-L1) not only significantly enriched the probiotic genera *Lactobacillus* and *Bacillus* in the gut of colitis-associated CRC mice but also significantly reduced the abundance of the pathogenic group *norank_f_RS-E47_termite_group* (26) (Fig. 8H2); (iii) compared to the NS group, the Combo group showed a significant enrichment of the beneficial gut bacteria *Parabacteroides* (27) and *Monoglobus*, as well as a significant reduction in the abundance of the harmful *genus Ruminococcaceae_UCG-009* (28) (Fig. 8H3); and (iv) when comparing the Combo + aPD-L1 group to the NS group, the treatment with Combo + aPD-L1 in colitis-associated CRC mice led to a significant decrease in the abundance of opportunistic pathogen *unclassified_f__Erysipelotrichaceae* in the gut (Fig. 8H4).

**Fig 9:**
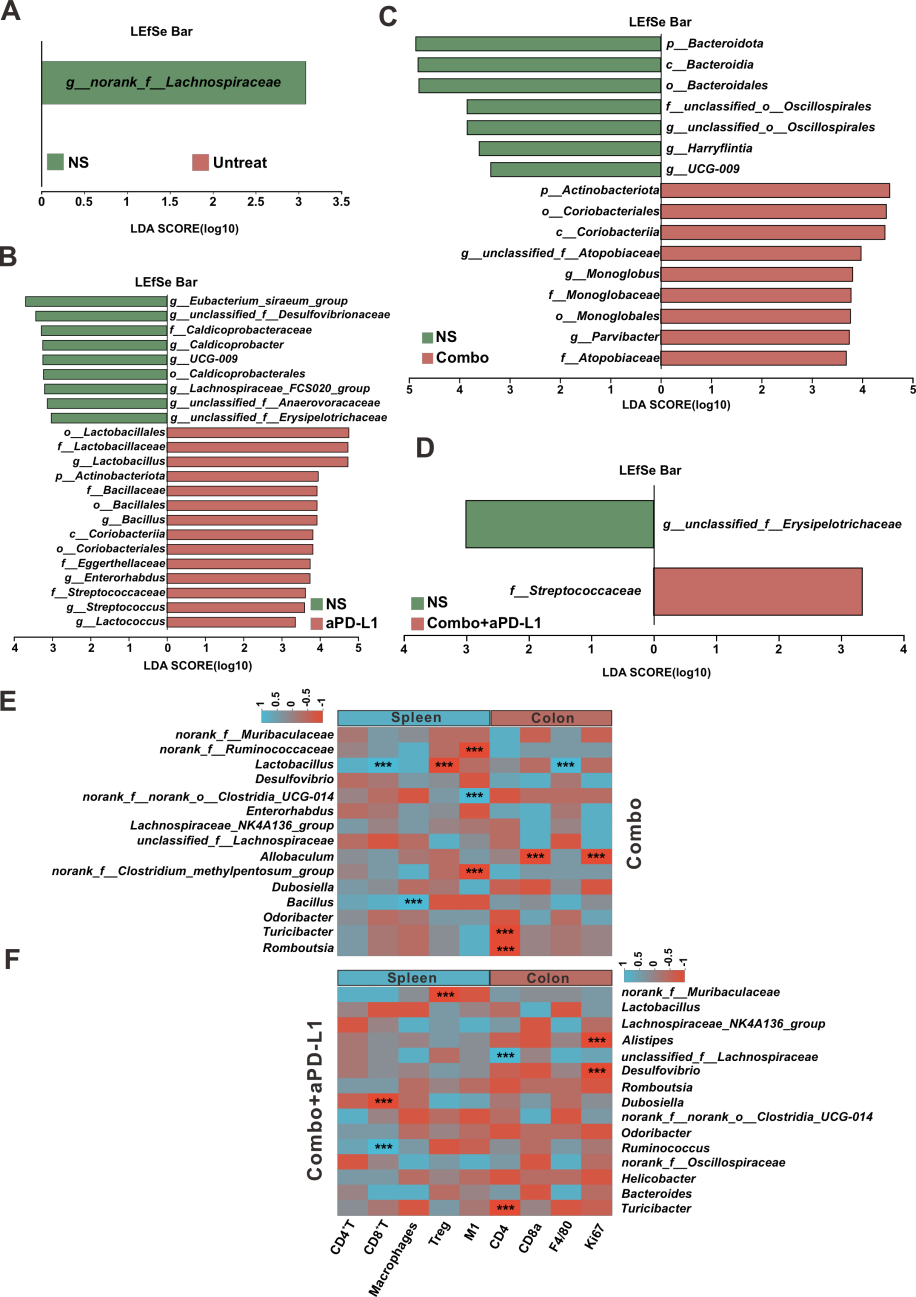
Using LEfSe and Spearman correlation analysis to investigate the intestinal microbiota in the colitis-associated CRC mice model. Analysis of the microbial taxa difference of the NS vs. Untreated group (**A**), NS group vs. aPD-L1 group (**B**), NS group vs. Combo group (**C**), and NS group vs. Combo + aPD-L1 group (**D**). Heatmap of Spearman correlation between intestinal microbiota and colitis-related phenotype in Combo group (**E**) and Combo + aPD-L1 group (**F**).

Finally, the LEfSe was employed to identify the predominant microorganisms significantly contributing to the observed intergroup differences. When the LDA score was greater than 3, the NS group exhibited a higher LDA score for *g__norank_f_Lachnospiraceae* than the Untreated group (Fig. 9A). Research has indicated that *Lachnospiraceae* is significantly increased in the gut of patients with ulcerative colitis, and this family is closely associated with recurrent disease, adverse reactions to anti-tumor necrosis factor therapy, and post-surgical recurrence in Crohn’s disease patients (29). Therefore, we speculate that the enriched *g__norank_f_Lachnospiraceae* in the gut of NS group mice may be related to the development and progression of colonic tumors. The LEfSe analysis comparing the NS and aPD-L1 groups revealed that the aPD-L1 group had higher LDA scores for *o__Lactobacillales, f__Lactobacillaceae, g__Lactobacillus, p__Actinobacteriota, f__Bacillaceae, o__Bacillales,* and *g__Bacillus* (Fig. 9B), which is consistent with the results in Fig. 8H.

In contrast to the NS group, the Combo group exhibited higher LDA scores for the beneficial bacteria *p__Actinobacteriota, g__Monoglobus,* and *g__Parvibacter* (Fig. 9C). For the Combo + aPD-L1 group, compared to the NS group, the Combo + aPD-L1 intervention not only effectively reduced the LDA score of *g__unclassified_f__Erysipelotrichaceae* but also significantly increased the abundance of the short-chain fatty acid-producing *f__Streptococcaceae* (Fig. 9D), which is consistent with the results in Fig. 8H. These findings suggest that the dominant microbial species identified through LEfSe analysis may largely reflect the differences in microbiota composition between the NS and Untreated groups, as well as among the NS group and other treatment groups (aPD-L1, Combo, and Combo + aPD-L1). These findings suggest that while there were no significant differences in bacteria composition among the Combo, Combo + aPD-L1, and NS groups, Combo or Combo + aPD-L1 intervention altered the abundance of certain bacterial species, such as *g__Monoglobus* and *g__unclassified_f__Erysipelotrichaceae*. These alterations may be associated with the observed improvements in colitis-associated CRC symptoms.

### Analysis of the correlation between clinical factors and gut microbiota in colitis-associated CRC mice

In this study, we selected the Combo group (13 W) and Combo + aPD-L1 group (13 W), demonstrating promising therapeutic effects in colitis-associated CRC mice for further analysis. We employed Spearman’s correlation analysis to explore the relationship between the gut microbiota of the above groups and clinical factors such as immune cells (CD4^+^ T, CD8^+^ T, macrophages, Treg, and M1 macrophage) in the spleen and the colon (Ki67, CD4^+^ T, CD8^+^ T, and macrophages [F4/80]), which measured using IHC staining. In the Combo group’s correlation analysis (Fig. 9E), *norank_f_Ruminococcaceae* was strongly negatively correlated with M1 macrophages in the spleen; *Lactobacillus* was positively correlated with CD8^+^ T cells in the spleen and colon (macrophages [F4/80]), and negatively correlated with Treg in the spleen. However, *Allobaculum* was strongly negatively correlated with CD8^+^ T cells and Ki67 in the colon, whereas *Turicibacter* and *Romboutsia* were negatively correlated with CD4^+^ T cells in the colon. In the Combo + aPD-L1 group’s correlation analysis (Fig. 9F), *norank_f_Muribaculaceae*, which has anti-inflammatory properties in the gut, was negatively correlated with Treg in the spleen; *Alistipes* and *Desulfovibrio* were negatively correlated with Ki67 in the colon; CD4^+^ T cells in the colon were positively correlated with *Unclassified_f_Lachnospiraceae* and negatively correlated with *Turicibacter*; CD8^+^ T cells in the spleen were negatively correlated with *Dubosiella* and positively correlated with *Ruminococcus*. In summary, the findings mentioned above elucidate that Combo or Combo + aPD-L1 intervention exerts anti-CRC effects by modulating the gut microbiome of colitis-associated CRC mice, specifically favoring the enrichment of certain microbial taxa, including *norank_f_Ruminococcaceae*, *Lactobacillus*, and *Allobaculum*. These alterations in the gut microbiota composition contribute to enhanced anti-CRC immune response in the mice.

### FMT of Con + aPD-L1’s FMTF or SFF significantly increases mice survival rate and reduces colon tumor counts

**Fig 10:**
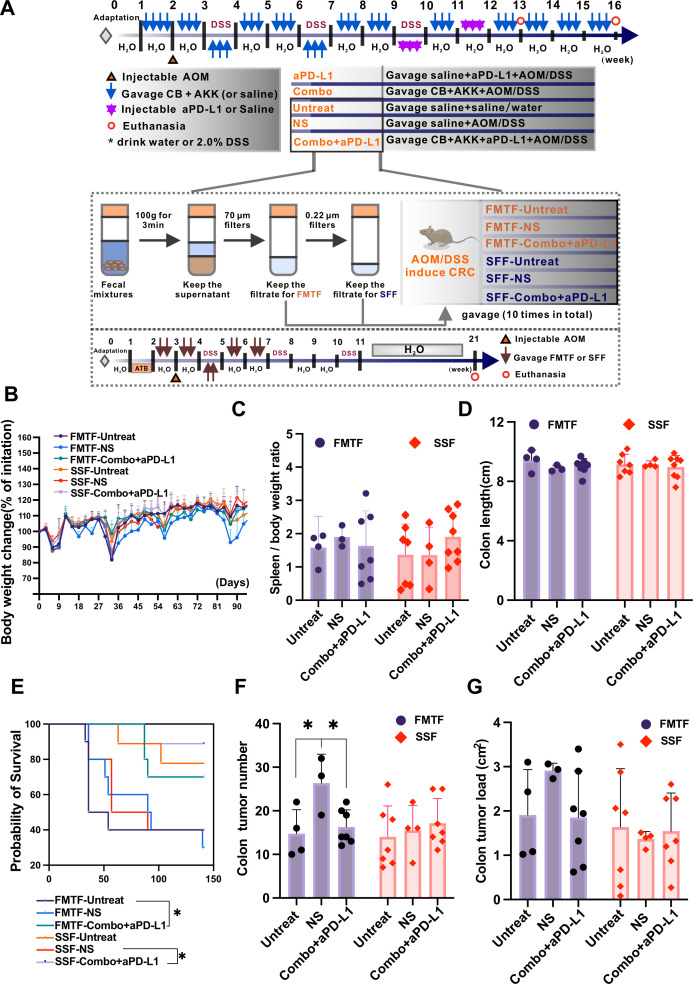
FMT of Combo-aPD-L1’s FMTF or SSF significantly improves the survival rate and reduces colon tumor counts. (**A**) Schematic representation of the colitis-associated CRC mouse model and FMT procedure. The blue arrows indicate that the mice were orally gavaged with bacteria. (**B**) Body weight change rate for mice in each group. (**C**) Spleen-to-body weight ratio for each group of mice. (**D**) Colon length. (**E**) Analysis of survival rates for each group of mice, with inter-group survival curves compared using the log-rank (Mantel-Cox) test, *P < 0.05, **P < 0.01, ***P < 0.001. (**F**) Colon tumor counts and (**G**) tumor area calculation in colitis-associated CRC mice. Data are presented as mean ± SD and were analyzed using one-way ANOVA with Tukey’s multiple comparison test. *P < 0.05, **P < 0.01, ***P < 0.001, compared with the FMTF-NS group; #*P* < 0.05, ##*P* < 0.01, ###*P* < 0.001, ####*P* < 0.0001, compared with the SFF-NS group.

We further explored the possible mechanisms by which Combo + aPD-L1 affects the colitis-associated CRC mice model. We transplanted the filtered microbiota transplantation fluid (FMTF) or sterile feces fluid (SSF) of Untreated, NS, and Combo + aPD-L1 mice into another group of colitis-associated CRC mice model (Fig. 10A) and observed their physiological and pathological changes. Although there were no statistically significant differences in body weight (Fig. 10B), spleen index (Fig. 10C), or colon length (Fig. 10D) among the groups after FMT and SSF treatment, it is noteworthy note that mice transplanted with FMTF-Combo + aPD-L1 and SSF-Combo + aPD-L1 had significantly improved survival rates compared with the FMTF-Untreated and SSF-NS groups (Fig. 10E). In addition, transplantation of FMTF-Untreated or FMTF-Combo + aPD-L1 groups mice feces significantly reduced the colon tumor number compared to transplanting feces from FMTF-NS group to colitis-associated CRC mice (Fig. 10F). Our findings demonstrate that fecal transplantation from Combo + aPD-L1-treated mice significantly reduced tumor burden and prolonged survival in AOM/DSS-induced mice. These results suggest that Combo + aPD-L1 intervention modulates the gut microbiome (microbial composition and metabolites) to suppress colitis-associated CRC progression, offering a promising novel therapeutic approach for CRC.

## DISCUSSION

Maintaining intestinal homeostasis relies on the mutual coordination between the gut microbiota, intestinal epithelial cells, and the host immune system (30, 31). The occurrence and development of intestinal diseases such as IBD and CRC are closely related to changes in intestinal homeostasis (1). Both CB and AKK have been proven to play multiple probiotic roles in intestinal diseases like IBD or CRC, including alleviating intestinal inflammation, regulating the stability of the gut microbiota, inhibiting tumor cell proliferation, promoting cancer cell apoptosis, and regulating the host’s anti-tumor immunity (13–16, 32). However, there are no reports on the efficacy and immune regulatory mechanisms of the combination of CB and AKK for IBD or colitis-associated CRC. In the present study, compared with single strains, the co-administration of CB and AKK not only significantly alleviated symptoms such as weight loss, colon shortening, and increased DAI in IBD mice but also regulated the disorder of intestinal microbiota composition in IBD mice, effectively reversed colonic inflammation in both IBD and colitis-associated CRC mice models. We also found that the combined administration of CB and AKK can enhance the sensitivity of colitis-associated CRC mice to the immune checkpoint inhibitor aPD-L1, significantly improving the efficacy of immunotherapy and the survival rate of *in situ* colitis-associated CRC mice. In addition, FMT therapy results showed that transplanting feces from colitis-associated CRC mice treated with combined CB and AKK into other colitis-associated CRC mice not only significantly reduced the formation of tumors in the colon but also significantly prolonged the survival rate of colitis-associated CRC mice. These results suggest that the combined use of two probiotics can potentially treat IBD and colitis-associated CRC.

Multiple studies have indicated that the intestines of colitis mice are prone to reduced mucus and increased permeability, which suggest intestinal barrier damage, and the expression levels and activity changes of proteins such as claudins, occludin, and ZO-1 (33, 34) can better reflect the state of intestinal barrier function (35). Previous studies have shown that CB or AKK can increase intestinal epithelium’s integrity and improve the disrupted intestinal barrier in acute colitis induced by DSS (16, 20). In the present study, we observed that intervention with single strains of CB or AKK could, to some extent, promote the expression of ZO-1 and occludin proteins in colonic tissue, consistent with previous studies (36–38). Whereas the combination of CB and AKK significantly promoted the expression of ZO-1 and occludin proteins in the colon, indicating that the combination of CB and AKK can better alleviate the damage to the colonic barrier function in IBD mice than single strains.

In addition, intestinal oxidative stress can disrupt the tight junction status between intestinal epithelial cells, leading to impaired intestinal epithelial barrier function and increased intestinal permeability, which may induce diseases such as GI diseases (9). Previous reports found that CB could reduce the levels of MPO (an enzyme reflecting the level of inflammation and oxidative stress) and MDA (the content of MDA can assess antioxidant activity) in the endometrial inflammation tissue of mice (39). In this study, we found that the combination of CB and AKK in IBD mice can more significantly reduce the levels of MDA and MPO in the serum or colon of mice than the single intervention of CB or AKK, indicating that the combination of CB and AKK can better alleviate the oxidative stress damage caused by DSS-induced colitis in IBD mice and improve the disrupted intestinal barrier. In IBD patients or DSS-induced IBD mice, intestinal epithelial tissue damage is prone to induce severe intestinal inflammatory immune responses (10). Many studies have shown that CB or AKK can play a protective role in mouse colitis by regulating some key components related to the inflammatory response, such as pro-inflammatory cytokines (IL-1β, IL-6, and TNF-α) and anti-inflammatory cytokines (IL-4 and IL-10) (12, 13, 40). In the present study, compared with the single intervention of CB or AKK in IBD, it was found that the combination of these two probiotics not only significantly promoted the release of anti-inflammatory factors IL-4 and IL-10 in the serum or colonic tissue but also significantly downregulated the expression of inflammatory factors such as IL-1β, IL-6, and TNF-α in the serum or colon, indicating that the combination of CB and AKK can better promote anti-inflammatory and inhibit the inflammatory response in the colonic tissue of IBD mice.

Probiotics can regulate the composition of the intestinal microbiota, thereby exerting a therapeutic effect on colonic inflammation (10). Compared with healthy mice, DSS induction can lead to a decrease in the α diversity of the gut microbiota in mice and changes in the composition of microbial communities (41). We found that compared with single-strain treatment, the combination of CB and AKK can significantly increase the α and β diversity of the fecal gut microbiota in IBD mice, which is in line with supplementing mice with CB or AKK can regulate intestinal microbiota disorder (12, 42). In previous reports, the relative abundance of probiotics (e.g., *Bifidobacterium* and *Lactobacillus*) was decreased in IBD patients and mice (43, 44). *Bifidobacterium* and *Lactobacillus* can enhance intestinal barrier function, regulating intestinal microbiota composition, host immunity, and anti-inflammatory and anticancer effects (44, 45). Interestingly, in the present study, after DSS induction, the *Lactobacillus* genus was significantly enriched in the gut IBD mice model compared with the untreated control group. Whereas, after intervention with combined CB and AKK, the abundance of *Lactobacillus* was reversed, which differs from previous reports. In addition, we also found that DSS induction may reduce the abundance level of *Bifidobacterium* in the gut. After the intervention with the combined CB and AKK, the relative abundance of *Bifidobacterium* in the gut can be significantly increased. Based on these results, we believe that although the *Lactobacillus* genus is generally considered a probiotic with a positive effect on human health, some specific strains of *Lactobacillus* may also have adverse effects under certain circumstances (46). In summary, we reasoned that DSS treatment disrupted the microbiota in the IBD mice’s gut. After administration of combined CB and AKK, the restored balance of the gut microbiota could be related to a significantly increased abundance of *Bifidobacterium* and decreased *Lactobacillus*.

Further analysis found that compared with the Untreated group, low-abundance *Lactobacilli* in the Combo group were significantly positively correlated with the levels of anti-inflammatory factors IL-4 and IL-10 in serum. By contrast, high-abundance *Bifidobacteria* were significantly negatively correlated with the levels of pro-inflammatory factor IL-6 and anti-inflammatory factor IL-4 in the colon. Previous studies have found that CB can alleviate intestinal inflammation by stimulating the host intestine to secrete IL-10, regulating the NF-κB pathway, and recruiting regulatory T cell (21). In conclusion, in the combined CB and AKK treatment group, low-abundance *Lactobacilli* significantly increased the levels of anti-inflammatory factors IL-4 and IL-10, and high-abundance *Bifidobacteria* reduced the level of pro-inflammatory factor IL-6, resulting in a significant improvement in colonic inflammation in IBD mice.

Probiotics have a variety of anti-tumor mechanisms, some of which exert anti-CRC effects by secreting protein derivatives (47–49) and others by producing small molecule compounds (50, 51). CB, the main butyrate producer in the human intestine, can inhibit tumor cell proliferation by acting as a histone deacetylase inhibitor and can also inhibit the development of colorectal tumors by downregulating the tumor WNT signaling pathway (22). Interestingly, AKK can promote the synthesis of butyrate in the distal colon tissue of the intestine (52, 53). Combining CB and AKK could significantly reduce tumor volume in the CRC mice model. The key mechanism of its anti-CRC therapeutic effect could be regulating microbiota changes in the intestine, thereby mediating the host’s anti-tumor immune response. In this study, our results showed that combining CB and AKK could better inhibit the abnormal proliferation of tumor cells in the colon.

CD8^+^ T cells are considered critical cells in anti-tumor immunity. In the tumor microenvironment, CD8^+^ T cells regulate the strength and duration of the immune response to avoid excessive immune reactions that damage the body. The activity and number of CD8^+^ T cells are closely related to tumor immune escape and patient prognosis (54, 55). Studies have shown that many microorganisms can stimulate CD8^+^ T-cell production to enhance host anti-tumor immunity, such as CB, AKK, *Lactobacillus reuteri, Roseburia intestinalis*, etc. (17, 51, 56, 57). Our results showed that the proportion of CD8^+^ T cells in NS group mice’s spleen and colon tissues was significantly higher than that of the Untreated group.

By contrast, the proportion of CD8^+^T cells in the CB and AKK combined intervention group showed a declining trend. This result is consistent with the report of Wang et al., who found that administration of pasteurized *A. muciniphila* or Amuc_1100 protein could significantly inhibit the occurrence of colon tumors in colitis-associated CRC mice (15), and the proportion of CD8^+^ T cells in the colon was significantly lower than that in the colitis-associated CRC control group. In the present study, we found that combination of CB and AKK moderately regulates the activation and duration of CD8^+^T cells, which could avoid excessive inflammatory immune response (58); thus, CD8^+^ T cells were better suited for exerting long-lasting anti-tumor effects.

In addition, macrophages participate in immune regulation through multiple mechanisms in innate immunity, including antigen clearance, antigen presentation, promoting or regulating inflammatory response, and promoting angiogenesis and immune escape in the tumor microenvironment (59). In previous studies, Nishimura et al. demonstrated that CD8^+^ T cells can promote the activation and recruitment of macrophages in inflammatory reactions (54), thereby aggravating the inflammatory response. Wang et al. (15) found that when AKK was used to treat colitis-associated CRC, the number of CD8^+^ T cells and macrophages in the spleen and colon tissues of mice was significantly reduced. We found that after CB and AKK combined treatment, the proportion of macrophages in the spleen and colon of colitis-associated CRC mice was significantly reduced. We speculate that CB and AKK can inhibit the recruitment and activation of macrophages by reducing CD8^+^ T cells, thereby inhibiting the inflammatory response in colitis-associated CRC and exerting anti-tumor effects.

Immune checkpoint inhibitors targeting the PD-1/PD-L1 axis are now widely used, and the composition of the gut microbiota in CRC patients is associated with the therapeutic efficacy of anti-PD-L1 monoclonal antibodies (24). Clinically, anti-PD-1 therapy fails to show significant therapeutic efficacy in 85% of CRC patients, and the “cold” tumor microenvironment in these patients limits the application of immunotherapy (60, 61). Studies have shown that many microorganisms can enhance the sensitivity of tumor mice to anti-PD-L1 immunotherapy, such as Lr, Ri, AKK, and CB (17, 51, 56, 57). In the present study, we found that anti-PD-L1 therapy in combination with CB and AKK effectively suppressed colitis-associated CRC tumor growth and reduced the over-activation of CD8^+^ T cells and macrophages in the inflammatory response. These results collectively suggest that combination of CB and AKK in CRC mice can not only directly exert anti-tumor effects but also enhance the sensitivity of colon tumor cells to aPD-L1, which could be a potential alternative strategy to treat “cold” CRC.

FMT is a promising therapeutic approach for various diseases. By modulating the composition and structure of the gut microbiota, FMT can improve the progression and symptoms of gut-related disorders. Studies have shown that transplanting healthy human gut microbiota into CRC mice can slow the progression of CRC in mice (62). We found that although Combo or Combo + aPD-L1 intervention did not significantly alter gut microbiota dysbiosis in colitis-associated CRC mice, transplantation of fecal microbiota from Combo + aPD-L1-treated mice suppressed colon tumor formation and prolonged the survival of colitis-associated CRC mice. Previously, Bertrand Routy et al. found that FMT combined with anti-PD-1 immunotherapy can effectively treat melanoma (63). The anti-CRC effect observed in the Combo group may be attributed to subtle alterations in the gut microbiome induced by CB and AKK interventions. The precise genetic underpinnings of these refined microbial compositional changes warrant further investigation.

Our study employing DSS-induced IBD and colitis-associated CRC mice models demonstrates that combining CB and AKK strains may exert their anti-inflammatory and anti-tumor effects by modulating gut microbiome dysbiosis. Notably, in the context of IBD, we observed superior anti-inflammatory efficacy of CB and AKK co-administration compared to single-strain therapy. Regarding the underlying therapeutic mechanisms in IBD and colitis-associated CRC, we hypothesize that (i) In IBD model, CB and AKK co-administration may restore the balance between *Bifidobacterium* and *Lactobacillus* genera in the gut, promoting the expression of anti-inflammatory cytokines IL-4 and IL-10, thereby effectively suppressing colonic inflammatory responses, and (ii) in colitis-associated CRC model, CB and AKK co-treatment may mitigate excessive infiltration of macrophages and CD8^+^ T cells in the colon, preventing an overactive immune response during anti-tumor therapy. In addition, regarding immune checkpoint inhibitors synergy, CB and AKK co-administration enhances the susceptibility of colitis-associated CRC mice to aPD-L1. Therefore, our findings further substantiate the potential of probiotic combination of CB and AKK for treating ulcerative colitis or colitis-associated CRC.

## MATERIALS AND METHODS

### Bacteria and culture condition

*Clostridium butyricum* CGMCC0313‐1 (CB) was anaerobically cultured at 37°C using the reinforced clostridium medium (RCM). The bacterial culture at OD_600_ = 0.6 was collected for subsequent experiments. *Akkermansia muciniphila* ATCC BAA-835 (AKK) was anaerobically cultured at 37°C using the thioglycollate medium. The bacterial culture at OD_600_ = 0.8 was collected for subsequent experiments.

### Construction of the IBD mouse model and combined intervention with CB and AKK

Thirty female C57BL/6L mice, aged 6–8 weeks and weighing (18 ± 2) g each, were chosen to perform the experiments (5 mice per group). They were housed in a 12/12 hour light/dark cycle and had *ad libitum* access to food and water in an environment with humidity (50% ± 5%) and temperature (22 ± 4°C). The mice were initially acclimated for 1 week. The Untreated group was not subjected to DSS treatment. By contrast, the treated groups were induced with acute colitis by administering drinking water containing 2.5% DSS (MW: 36–50 kDa, Cat# 60316ES25 & 60316ES60, Yeasen Biotech Co, Ltd, Shanghai, China) for 7 consecutive days (NS, CB, AKK, and Combo groups). In the DSS-induced colitis groups, each mouse was administered 100 µL of bacterial solution or saline solution by gavage every other day (CB group: OD_600_ = 0.6, 1.5 × 10^8^ CFU/mL; AKK group: OD_600_ = 0.8, 1.5 × 10^9^ CFU/mL; Combo group: mice were given 50 µL CB [OD = 1.2, 3.0 × 10^8^ CFU/mL] and 50 µL AKK [OD_600_ = 1.6, 3.0 × 10^9^]) bacterial suspension (specifically, removed the supernatant from bacterial cultures and then washed the bacterial pellet thoroughly with phosphate-buffered saline [PBS] to eliminate any residual supernatant components). The washed bacterial pellet was then collected for subsequent experiments (NS group: received an equal volume of normal saline [NS] treatment). The Untreated group gavaged 100 µL of NS to each mouse once/2 days. Mouse body weight, fecal characteristics, and occult blood were recorded daily, and the disease activity index (DAI) (15) for mice was calculated and recorded in Table S1.

### Construction of the AOM/DSS-induced colon cancer (colitis-associated CRC) mouse model and combined intervention with CB and AKK

Female C57BL/6L mice aged 6–8 weeks (at least 7 mice per group) were housed in a 12/12 hour light/dark cycle and had *ad libitum* access to food and water in an environment with humidity (50% ± 5%) and temperature (22°C ± 4°C). After acclimation for 1 week, mice weighing (20 ± 2) g were selected for AOM/DSS-induced colon cancer modeling, the molding process is outlined follows. (i) Every 3 days, the experimental groups were administered 100 µL of bacterial-suspension solution (Combo group: mice were given 50 µL CB [OD = 1.2, 3.0 × 10^8^ CFU/mL] and 50 µl AKK [OD_600_ = 1.6, 3.0 × 109 CFU/mL]) by gavage, and the Combo + aPD-L1 group received six intraperitoneal injections of Anti-PD-L1 mAb (aPD-L1, RecombiMAb anti-mouse PD-L1 [B7-H1] [LALA-PG, MedImmune, RRID: AB_10949073, Cat# BE0101-50mg]) at different time points, while the other groups received six intraperitoneal injections of NS at the same time points. (ii) On day 1, the mice were weighed and noted, and AOM (10 mg/kg, MedChemExpress, Cat#: hy-111375) was intraperitoneally injected in the experimental group. (iii) Mice were fed with regular drinking water for one week. (iv) Mice were fed with 2.0% DSS in drinking water for one week. (v) Mice were fed with regular drinking water for 2 weeks. (vi) Repeat steps iii and iv three times. After treatment ii, each mouse’s fecal characteristics and occult blood were recorded; the mouse’s body weight was recorded every 3 days, and the disease activity index (DAI) was calculated. At the end of the modeling, the mice were anesthetized using isoflurane in a chamber at the 13th week (13W) and 16th week (16W), followed by euthanasia.

### Fecal microbiota transplantation (FMT) in colitis-associated CRC mice

Feces from the Untreated, NS, and Combo + aPD-L1 groups were collected and frozen. For transplantation, 100 mg/mL of feces from each group was centrifuged for 5 minutes at 100 × *g*. The supernatant was filtered through a 70 µm filter (Cat#CSS013070, Guangzhou Jet Bio-Filtration Co., Ltd., Guangzhou, China) to obtain Filtered Microbiota Transplantation Fluid (FMTF). TTRIzol filtered through a 0.22 µm bacterial filter (Cat#FPE204030, Guangzhou Jet Bio-Filtration Co., Ltd., Guangzhou, China) to obtain sterile fecal fluid (SFF) for subsequent experiments. Six- to eight-week-old female C57BL/6L mice (at least 9 mice per group) were housed under a 12/12 hour light/dark cycle. The mice were allowed free access to food and water in a humidity-controlled (50% ± 5%) and temperature-controlled (22 ± 4°C) environment. After one week of acclimation, mice weighing (20 ± 2) g were used for AOM/DSS-induced colon cancer modeling. The modeling procedure is as follows. (i) All groups of mice were given water containing ampicillin (1 g/L), vancomycin (0.5 g/L), metronidazole (1 g/L), and neomycin sulfate (1 g/L) for 1 week. (ii) At the end of the first week, the mice were switched to normal water. From the second week to the seventh week, the mice were given two doses of fecal microbiota transplantation per week (100 µL/mouse), for a total of 10 transplantations. (iii) At the third week, the mice in all groups were injected intraperitoneally with AOM at a dose of 10 mg/kg. The mice were then given normal water for 1 week. (iv) The mice were given normal water for 2 weeks. (v) The mice were given 2.0% DSS in their drinking water for 1 week. (vi) The mice were given normal water for 2 weeks. (vii) Steps iii and iv were repeated three times (17, 64). The mice’s body weight was recorded every 3 days. The mice were euthanized at 21 weeks, and fecal samples, spleen samples, and colon samples were collected simultaneously.

### High-throughput sequencing of 16S rRNA genes

Total bacterial DNA was extracted from mouse fecal samples using a fecal microbiome DNA extraction kit (Lot# Y1904, Tiangen Biotech (Beijing) Co., Ltd., Beijing, China). The V3 hypervariable region of the 16S rRNA gene was amplified using the primers 338F (5′-ACTCCTACGGGAGGCAGCA-3′) and 806R (5′-GGACTACHVGGGTWTCTAAT-3′). Sequencing was performed on an Illumina MiSeq platform (PE300, San Diego, CA, USA). Alpha diversity, richness, and sparsity curves were analyzed using operational taxonomic units (OTUs) with a threshold of 0.97 (65). In addition, partial least squares discriminant analysis (PLS-DA) was performed based on OTUs using the Bray-Curtis distance. All data analyses were performed on the Majorbio Cloud Platform (https://cloud.majorbio.com). Specifically, Alpha diversity indices, such as Chao 1 and Shannon index, were calculated using Mothur software (version 1.30.2, http://www.mothur.org/wiki/Calculators). Alpha diversity was analyzed for intergroup differences using the Wilcoxon rank-sum test. PCoA based on Bray-Curtis distance was used to test the similarity of microbial community structures between samples, and permutational multivariate analysis of variance (PERMANOVA) non-parametric test was used to analyze whether the differences in microbial community structures between sample groups were significant. Linear discriminant analysis Effect Size (LEfSe analysis) (http://huttenhower.sph.harvard.edu/lefse) (LDA > 3, *P* < 0.05) was used to identify bacterial taxa with significantly different abundances at the phylum to genus level between different groups. Species were selected for correlation network analysis based on Spearman correlation |*r*| > 0.6, *P* <0.05.

### Enzyme-linked immunosorbent assay

Mouse colon tissue was homogenized in a high-speed refrigerated tissue homogenizer (KZ-III-FP, Wuhan Servicebio Technology Co., Ltd, Wuhan, China). Protein concentration in the homogenate was determined using a BCA protein assay kit (Cat# PC0020, Beijing Solarbio Science & Technology Co., Ltd., Beijing, China). IL-4, IL-6, IL-10, IL-1β, TNF-α, myeloperoxidase (MPO), and malondialdehyde (MDA) levels in mouse colon tissue were measured using enzyme-linked immunosorbent assay (ELISA) kits (Cat# ml064310, ml098430, mIC50274-1, mIC50300-1, mIC50536-1, ml002070, ml077384, Shanghai Enzyme Linked Biotechnology Co., Ltd., Shanghai, China).

### Histology and immunohistochemistry staining

Colon tissue sections and immunohistochemistry (IHC) staining were performed by Wuhan Huayan Biotechnology Co., Ltd., using H&E staining. IBD mice were stained with zonula occludens-1 (ZO-1) and occludin by IHC. IHC was used to detect Ki67, caspase 3, CD4, CD8a, and F4/80 in the colon tissues of CRC mice. IBD mouse pathological scoring standards are shown in Table S2, and CRC mouse H&E pathological scoring are shown in Table S3. IHC staining results were analyzed and processed using Image J (Fiji for Mac OS X, U.S. National Institutes of Health, Bethesda, MD).

### Flow cytometry

The spleens of each group of mice were obtained after the modeling was completed to analyze the changes in immune factors in the spleen of colitis-associated CRC mice. The spleens were gently homogenized in PBS (Cat#BC20230508, Bio-Channel Biotechnology Co., Ltd, Nanjing, China), and the single-cell suspension of the spleen was obtained after filtering through a 100 µm cell sieve. After lysing the red blood cells in the suspension, the spleen cells were stained with fluorescently labeled antibodies (Table S4). The antibodies for analyzing lymphocyte subsets included the following: APC-Cy7 rat anti-mouse CD45 (Cat# 557659, RRID: AB_396774, Becton, Dickinson and Company, CA, USA), FITC hamster anti-mouse CD3e (Cat#553061, RRID: AB_394595, Becton, Dickinson and Company, CA, USA), APC/BV605 rat anti-mouse CD4 (Cat#563151, RRID: AB_398528/AB_2687549, Becton, Dickinson and Company, CA, USA), PerCP-CY5.5 rat anti-mouse CD8a (Cat#551162, RRID: AB_394081, Becton, Dickinson and Company, CA, USA), and BV650 rat anti-mouse CD25 (Cat#552880, RRID: AB_2738547, Becton, Dickinson and Company, CA, USA). Macrophages were analyzed by staining spleen cells with fluorescently labeled antibodies. The antibodies included FITC rat anti-CD11b (Cat#553310, RRID: AB_396679, Becton, Dickinson and Company, CA, USA), PE rat anti-mouse CD86 (Cat#553692, RRID: AB_394994, Becton, Dickinson and Company, CA, USA), and APC rat anti-mouse F4/80 (Cat#566787, RRID: AB_2869866, Becton, Dickinson and Company, CA, USA). All samples were analyzed using BD-FACSCelesta (BD FACSCelesta, Becton, Dickinson, and Company, CA, USA), and the data were analyzed using FlowJo software (version 10, Becton, Dickinson and Company, CA, USA). Gating strategies for FCM are shown in Fig. S3.

### Data and statistical analysis

All clinical samples and animal data in this study were analyzed. Two-group comparisons were performed using Students’ *t*-tests or non-parametric tests. Multiple-group comparisons were performed using one-way ANOVA followed by Tukey’s multiple comparisons. When the characteristics of the sample data meet the assumption of normal distribution, one-way ANOVA is used. If the normal distribution assumption is not satisfied, the Kruskal-Wallis test is used. All figures were presented as mean ± standard deviation. A *P*-value < 0.05 was considered statistically significant. All statistical analyses were performed using GraphPad Prism software (version 9.0, GraphPad Software, San Diego, CA).

## Data Availability

All sequencing data were uploaded to the NCBI Sequence Read Archive (https://www.ncbi.nlm.nih.gov/bioproject/PRJNA1135783/) with the accession number PRJNA1135783 (BioProject).

## SUPPLEMENTAL MATERIAL

Supplemental material msystems.01567-24-s0001.docx Supplemental tables and figures.

ASM does not own the copyrights to Supplemental Material that may be linked to, or accessed through, an article. The authors have granted ASM a non-exclusive, world-wide license to publish the Supplemental Material files. Please contact the corresponding author directly for reuse.

## References

[1] Danne C, Skerniskyte J, Marteyn B, Sokol H. 2024. Neutrophils: from IBD to the gut microbiota. Nat Rev Gastroenterol Hepatol 21:184–197. doi:10.1038/s41575-023-00871-3 38110547

[2] Lavelle A, Sokol H. 2020. Gut microbiota-derived metabolites as key actors in inflammatory bowel disease. Nat Rev Gastroenterol Hepatol 17:223–237. doi:10.1038/s41575-019-0258-z 32076145

[3] Praveschotinunt P, Duraj-Thatte AM, Gelfat I, Bahl F, Chou DB, Joshi NS. 2019. Engineered E. coli Nissle 1917 for the delivery of matrix-tethered therapeutic domains to the gut. Nat Commun 10:5580. doi:10.1038/s41467-019-13336-6 31811125 PMC6898321

[4] Zhang S, Ermann J, Succi MD, Zhou A, Hamilton MJ, Cao B, Korzenik JR, Glickman JN, Vemula PK, Glimcher LH, Traverso G, Langer R, Karp JM. 2015. An inflammation-targeting hydrogel for local drug delivery in inflammatory bowel disease. Sci Transl Med 7:300ra128. doi:10.1126/scitranslmed.aaa5657 PMC4825054 26268315

[5] Rosen MJ, Dhawan A, Saeed SA. 2015. Inflammatory bowel disease in children and adolescents. JAMA Pediatr 169:1053–1060. doi:10.1001/jamapediatrics.2015.1982 26414706 PMC4702263

[6] Shah SC, Itzkowitz SH. 2022. Colorectal cancer in inflammatory bowel disease: mechanisms and management. Gastroenterology 162:715–730. doi:10.1053/j.gastro.2021.10.035 34757143 PMC9003896

[7] Li N, Lu B, Luo C, Cai J, Lu M, Zhang Y, Chen H, Dai M. 2021. Incidence, mortality, survival, risk factor and screening of colorectal cancer: a comparison among China, Europe, and northern America. Cancer Lett 522:255–268. doi:10.1016/j.canlet.2021.09.034 34563640

[8] Dudley JC, Lin M-T, Le DT, Eshleman JR. 2016. Microsatellite instability as a biomarker for PD-1 blockade. Clin Cancer Res 22:813–820. doi:10.1158/1078-0432.CCR-15-1678 26880610

[9] Leonard MM, Valitutti F, Karathia H, Pujolassos M, Kenyon V, Fanelli B, Troisi J, Subramanian P, Camhi S, Colucci A, Serena G, Cucchiara S, Trovato CM, Malamisura B, Francavilla R, Elli L, Hasan NA, Zomorrodi AR, Colwell R, Fasano A, CD-GEMM Team. 2021. Microbiome signatures of progression toward celiac disease onset in at-risk children in a longitudinal prospective cohort study. Proc Natl Acad Sci U S A 118:e2020322118. doi:10.1073/pnas.2020322118 34253606 PMC8307711

[10] Tilg H, Adolph TE, Gerner RR, Moschen AR. 2018. The intestinal microbiota in colorectal cancer. Cancer Cell 33:954–964. doi:10.1016/j.ccell.2018.03.004 29657127

[11] Zhao Z, Ning J, Bao X-Q, Shang M, Ma J, Li G, Zhang D. 2021. Fecal microbiota transplantation protects rotenone-induced Parkinson’s disease mice via suppressing inflammation mediated by the lipopolysaccharide-TLR4 signaling pathway through the microbiota-gut-brain axis. Microbiome 9:226. doi:10.1186/s40168-021-01107-9 34784980 PMC8597301

[12] Ma L, Shen Q, Lyu W, Lv L, Wang W, Yu M, Yang H, Tao S, Xiao Y. 2022. Clostridium butyricum and its derived extracellular vesicles modulate gut homeostasis and ameliorate acute experimental colitis. Microbiol Spectr 10:e01368-22. doi:10.1128/spectrum.01368-22 35762770 PMC9431305

[13] Pu W, Zhang H, Zhang T, Guo X, Wang X, Tang S. 2023. Inhibitory effects of Clostridium butyricum culture and supernatant on inflammatory colorectal cancer in mice. Front Immunol 14:1004756. doi:10.3389/fimmu.2023.1004756 37081884 PMC10111964

[14] Xu H, Luo H, Zhang J, Li K, Lee M-H. 2023. Therapeutic potential of Clostridium butyricum anticancer effects in colorectal cancer. Gut Microbes 15:2186114. doi:10.1080/19490976.2023.2186114 36941257 PMC10038047

[15] Wang L, Tang L, Feng Y, Zhao S, Han M, Zhang C, Yuan G, Zhu J, Cao S, Wu Q, Li L, Zhang Z. 2020. A purified membrane protein from Akkermansia muciniphila or the pasteurised bacterium blunts colitis associated tumourigenesis by modulation of CD8^+^ T cells in mice. Gut 69:1988–1997. doi:10.1136/gutjnl-2019-320105 32169907 PMC7569398

[16] Meynier M, Daugey V, Mallaret G, Gervason S, Meleine M, Barbier J, Aissouni Y, Lolignier S, Bonnet M, Ardid D, De Vos WM, Van Hul M, Suenaert P, Brochot A, Cani PD, Carvalho FA. 2024. Pasteurized Akkermansia muciniphila improves irritable bowel syndrome-like symptoms and related behavioral disorders in mice. Gut Microbes 16:2298026. doi:10.1080/19490976.2023.2298026 38170633 PMC10766393

[17] Kang X, Liu C, Ding Y, Ni Y, Ji F, Lau HCH, Jiang L, Sung JJ, Wong SH, Yu J. 2023. Roseburia intestinalis generated butyrate boosts anti-PD-1 efficacy in colorectal cancer by activating cytotoxic CD8^+^ T cells. Gut 72:2112–2122. doi:10.1136/gutjnl-2023-330291 37491158 PMC10579466

[18] Bell HN, Rebernick RJ, Goyert J, Singhal R, Kuljanin M, Kerk SA, Huang W, Das NK, Andren A, Solanki S, Miller SL, Todd PK, Fearon ER, Lyssiotis CA, Gygi SP, Mancias JD, Shah YM. 2022. Reuterin in the healthy gut microbiome suppresses colorectal cancer growth through altering redox balance. Cancer Cell 40:185–200. doi:10.1016/j.ccell.2021.12.001 34951957 PMC8847337

[19] Salvi PS, Cowles RA. 2021. Butyrate and the intestinal epithelium: modulation of proliferation and inflammation in homeostasis and disease. Cells 10:1775. doi:10.3390/cells10071775 34359944 PMC8304699

[20] Liu M, Xie W, Wan X, Deng T. 2020. Clostridium butyricum modulates gut microbiota and reduces colitis associated colon cancer in mice. Int Immunopharmacol 88:106862. doi:10.1016/j.intimp.2020.106862 32771947

[21] Zhou M, Yuan W, Yang B, Pei W, Ma J, Feng Q. 2022. Clostridium butyricum inhibits the progression of colorectal cancer and alleviates intestinal inflammation via the myeloid differentiation factor 88 (MyD88)-nuclear factor-kappa B (NF-κB) signaling pathway. Ann Transl Med 10:478. doi:10.21037/atm-22-1670 35571406 PMC9096358

[22] Chen D, Jin D, Huang S, Wu J, Xu M, Liu T, Dong W, Liu X, Wang S, Zhong W, Liu Y, Jiang R, Piao M, Wang B, Cao H. 2020. Clostridium butyricum, a butyrate-producing probiotic, inhibits intestinal tumor development through modulating Wnt signaling and gut microbiota. Cancer Lett 469:456–467. doi:10.1016/j.canlet.2019.11.019 31734354

[23] Plovier H, Everard A, Druart C, Depommier C, Van Hul M, Geurts L, Chilloux J, Ottman N, Duparc T, Lichtenstein L, Myridakis A, Delzenne NM, Klievink J, Bhattacharjee A, van der Ark KCH, Aalvink S, Martinez LO, Dumas M-E, Maiter D, Loumaye A, Hermans MP, Thissen J-P, Belzer C, de Vos WM, Cani PD. 2017. A purified membrane protein from Akkermansia muciniphila or the pasteurized bacterium improves metabolism in obese and diabetic mice. Nat Med 23:107–113. doi:10.1038/nm.4236 27892954

[24] Routy B, Le Chatelier E, Derosa L, Duong CPM, Alou MT, Daillère R, Fluckiger A, Messaoudene M, Rauber C, Roberti MP, et al.. 2018. Gut microbiome influences efficacy of PD-1-based immunotherapy against epithelial tumors. Science 359:91–97. doi:10.1126/science.aan3706 29097494

[25] Depommier C, Everard A, Druart C, Plovier H, Van Hul M, Vieira-Silva S, Falony G, Raes J, Maiter D, Delzenne NM, de Barsy M, Loumaye A, Hermans MP, Thissen J-P, de Vos WM, Cani PD. 2019. Supplementation with Akkermansia muciniphila in overweight and obese human volunteers: a proof-of-concept exploratory study. Nat Med 25:1096–1103. doi:10.1038/s41591-019-0495-2 31263284 PMC6699990

[26] Shao J, Li Z, Gao Y, Zhao K, Lin M, Li Y, Wang S, Liu Y, Chen L. 2021. Construction of a “bacteria-metabolites” co-expression network to clarify the anti-ulcerative colitis effect of flavonoids of Sophora flavescens aiton by regulating the “host-microbe” interaction. Front Pharmacol 12:710052. doi:10.3389/fphar.2021.710052 34721011 PMC8553221

[27] Wang K, Liao M, Zhou N, Bao L, Ma K, Zheng Z, Wang Y, Liu C, Wang W, Wang J, Liu S-J, Liu H. 2019. Parabacteroides distasonis alleviates obesity and metabolic dysfunctions via production of succinate and secondary bile acids. Cell Rep 26:222–235. doi:10.1016/j.celrep.2018.12.028 30605678

[28] Wang Y, Yao W, Li B, Qian S, Wei B, Gong S, Wang J, Liu M, Wei M. 2020. Nuciferine modulates the gut microbiota and prevents obesity in high-fat diet-fed rats. Exp Mol Med 52:1959–1975. doi:10.1038/s12276-020-00534-2 33262480 PMC8080667

[29] Schirmer M, Garner A, Vlamakis H, Xavier RJ. 2019. Microbial genes and pathways in inflammatory bowel disease. Nat Rev Microbiol 17:497–511. doi:10.1038/s41579-019-0213-6 31249397 PMC6759048

[30] Zhu Z, Huang J, Li X, Xing J, Chen Q, Liu R, Hua F, Qiu Z, Song Y, Bai C, Mo Y-Y, Zhang Z. 2020. Gut microbiota regulate tumor metastasis via circRNA/miRNA networks. Gut Microbes 12:1788891. doi:10.1080/19490976.2020.1788891 32686598 PMC7524358

[31] Bertocchi A, Carloni S, Ravenda PS, Bertalot G, Spadoni I, Lo Cascio A, Gandini S, Lizier M, Braga D, Asnicar F, et al.. 2021. Gut vascular barrier impairment leads to intestinal bacteria dissemination and colorectal cancer metastasis to liver. Cancer Cell 39:708–724. doi:10.1016/j.ccell.2021.03.004 33798472

[32] Bian X, Wu W, Yang L, Lv L, Wang Q, Li Y, Ye J, Fang D, Wu J, Jiang X, Shi D, Li L. 2019. Administration of Akkermansia muciniphila ameliorates dextran sulfate sodium-induced ulcerative colitis in mice. Front Microbiol 10:2259. doi:10.3389/fmicb.2019.02259 31632373 PMC6779789

[33] Yang Y, Zhang Y, Song J, Li Y, Zhou L, Xu H, Wu K, Gao J, Zhao M, Zheng Y. 2023. Bergamot polysaccharides relieve DSS-induced ulcerative colitis via regulating the gut microbiota and metabolites. Int J Biol Macromol 253:127335. doi:10.1016/j.ijbiomac.2023.127335 37820919

[34] Zhu L, Yu T, Wang W, Xu T, Geng W, Li N, Zan X. 2024. Responsively degradable nanoarmor‐assisted super resistance and stable colonization of probiotics for enhanced inflammation‐targeted delivery. Adv Mater Weinheim 36:e2308728. doi:10.1002/adma.202308728 38241751

[35] Tajik N, Frech M, Schulz O, Schälter F, Lucas S, Azizov V, Dürholz K, Steffen F, Omata Y, Rings A, et al.. 2020. Targeting zonulin and intestinal epithelial barrier function to prevent onset of arthritis. Nat Commun 11:1995. doi:10.1038/s41467-020-15831-7 32332732 PMC7181728

[36] Huang X, Hu J, Zhang H, Li J, Zhu X, Liu Y, Liang Y, Mei Y. 2023. Clostridium butyricum and chitooligosaccharides in synbiotic combination ameliorate symptoms in a DSS-induced ulcerative colitis mouse model by modulating gut microbiota and enhancing intestinal barrier function. Microbiol Spectr 11:e04370-22. doi:10.1128/spectrum.04370-22 36975838 PMC10100383

[37] Li W, Zhang Y, Chen M, Guo X, Ding Z. 2024. The antioxidant strain Lactiplantibacillus plantarum AS21 and Clostridium butyricum ameliorate DSS-induced colitis in mice by remodeling the assembly of intestinal microbiota and improving gut functions. Food Funct 15:2022–2037. doi:10.1039/d3fo05337g 38289370

[38] He K-Y, Lei X-Y, Wu D-H, Zhang L, Li J-Q, Li Q-T, Yin W-T, Zhao Z-L, Liu H, Xiang X-Y, Zhu L-J, Cui C-Y, Wang K-K, Wang J-H, Lv L, Sun Q-H, Liu G-L, Xu Z-X, Jian Y-P. 2023. Akkermansia muciniphila protects the intestine from irradiation-induced injury by secretion of propionic acid. Gut Microbes 15:2293312. doi:10.1080/19490976.2023.2293312 38087436 PMC10730217

[39] Hagihara M, Ariyoshi T, Eguchi S, Oka K, Takahashi M, Kato H, Shibata Y, Umemura T, Mori T, Miyazaki N, Hirai J, Asai N, Mori N, Mikamo H. 2024. Oral Clostridium butyricum on mice endometritis through uterine microbiome and metabolic alternations. Front Microbiol 15:1351899. doi:10.3389/fmicb.2024.1351899 38450161 PMC10915095

[40] Xi M, Li J, Hao G, An X, Song Y, Wei H, Ge W. 2020. Stachyose increases intestinal barrier through Akkermansia muciniphila and reduces gut inflammation in germ-free mice after human fecal transplantation. Food Res Int 137:109288. doi:10.1016/j.foodres.2020.109288 33233042

[41] Wu Z, Huang S, Li T, Li N, Han D, Zhang B, Xu ZZ, Zhang S, Pang J, Wang S, Zhang G, Zhao J, Wang J. 2021. Gut microbiota from green tea polyphenol-dosed mice improves intestinal epithelial homeostasis and ameliorates experimental colitis. Microbiome 9:184. doi:10.1186/s40168-021-01115-9 34493333 PMC8424887

[42] Liu Y, Yang M, Tang L, Wang F, Huang S, Liu S, Lei Y, Wang S, Xie Z, Wang W, Zhao X, Tang B, Yang S. 2022. TLR4 regulates RORγT^+^ regulatory T-cell responses and susceptibility to colon inflammation through interaction with Akkermansia muciniphila. Microbiome 10:98. doi:10.1186/s40168-022-01296-x 35761415 PMC9235089

[43] Cox SR, Lindsay JO, Fromentin S, Stagg AJ, McCarthy NE, Galleron N, Ibraim SB, Roume H, Levenez F, Pons N, Maziers N, Lomer MC, Ehrlich SD, Irving PM, Whelan K. 2020. Effects of low FODMAP diet on symptoms, fecal microbiome, and markers of inflammation in patients with quiescent inflammatory bowel disease in a randomized trial. Gastroenterology 158:176–188. doi:10.1053/j.gastro.2019.09.024 31586453

[44] Wu J, Wei Z, Cheng P, Qian C, Xu F, Yang Y, Wang A, Chen W, Sun Z, Lu Y. 2020. Rhein modulates host purine metabolism in intestine through gut microbiota and ameliorates experimental colitis. Theranostics 10:10665–10679. doi:10.7150/thno.43528 32929373 PMC7482825

[45] Mandelbaum N, Zhang L, Carasso S, Ziv T, Lifshiz-Simon S, Davidovich I, Luz I, Berinstein E, Gefen T, Cooks T, Talmon Y, Balskus EP, Geva-Zatorsky N. 2023. Extracellular vesicles of the Gram-positive gut symbiont Bifidobacterium longum induce immune-modulatory, anti-inflammatory effects. NPJ Biofilms Microbiomes 9:30. doi:10.1038/s41522-023-00400-9 37270554 PMC10239484

[46] Hezaveh K, Shinde RS, Klötgen A, Halaby MJ, Lamorte S, Ciudad MT, Quevedo R, Neufeld L, Liu ZQ, Jin R, et al.. 2022. Tryptophan-derived microbial metabolites activate the aryl hydrocarbon receptor in tumor-associated macrophages to suppress anti-tumor immunity. Immunity 55:324–340. doi:10.1016/j.immuni.2022.01.006 35139353 PMC8888129

[47] Chen X, Li P, Luo B, Song C, Wu M, Yao Y, Wang D, Li X, Hu B, He S, Zhao Y, Wang C, Yang X, Hu J. 2024. Surface mineralization of engineered bacterial outer membrane vesicles to enhance tumor photothermal/immunotherapy. ACS Nano 18:1357–1370. doi:10.1021/acsnano.3c05714 38164903

[48] Ma L, Lyu W, Song Y, Chen K, Lv L, Yang H, Wang W, Xiao Y. 2023. Anti-inflammatory effect of Clostridium butyricum-derived extracellular vesicles in ulcerative colitis: impact on host micrornas expressions and gut microbiome profiles. Mol Nutr Food Res 67:e2200884. doi:10.1002/mnfr.202370029 37183784

[49] Zhang YL, Lü R, Chang ZS, Zhang WQ, Wang QB, Ding SY, Zhao W. 2014. Clostridium sporogenes delivers interleukin-12 to hypoxic tumours, producing antitumour activity without significant toxicity. Lett Appl Microbiol 59:580–586. doi:10.1111/lam.12322 25163827

[50] Fong W, Li Q, Ji F, Liang W, Lau HCH, Kang X, Liu W, To K-W, Zuo Z, Li X, Zhang X, Sung JJ, Yu J. 2023. Lactobacillus gallinarum-derived metabolites boost anti-PD1 efficacy in colorectal cancer by inhibiting regulatory T cells through modulating IDO1/Kyn/AHR axis. Gut 72:2272–2285. doi:10.1136/gutjnl-2023-329543 37770127 PMC10715476

[51] Bender MJ, McPherson AC, Phelps CM, Pandey SP, Laughlin CR, Shapira JH, Medina Sanchez L, Rana M, Richie TG, Mims TS, Gocher-Demske AM, Cervantes-Barragan L, Mullett SJ, Gelhaus SL, Bruno TC, Cannon N, McCulloch JA, Vignali DAA, Hinterleitner R, Joglekar AV, Pierre JF, Lee STM, Davar D, Zarour HM, Meisel M. 2023. Dietary tryptophan metabolite released by intratumoral Lactobacillus reuteri facilitates immune checkpoint inhibitor treatment. Cell 186:1846–1862. doi:10.1016/j.cell.2023.03.011 37028428 PMC10148916

[52] Belzer C, Chia LW, Aalvink S, Chamlagain B, Piironen V, Knol J, de Vos WM. 2017. Microbial metabolic networks at the mucus layer lead to diet-independent butyrate and vitamin B_12_ production by intestinal symbionts. mBio 8:e00770-17. doi:10.1128/mBio.00770-17 28928206 PMC5605934

[53] Chia LW, Hornung BVH, Aalvink S, Schaap PJ, de Vos WM, Knol J, Belzer C. 2018. Deciphering the trophic interaction between Akkermansia muciniphila and the butyrogenic gut commensal Anaerostipes caccae using a metatranscriptomic approach. Antonie Van Leeuwenhoek 111:859–873. doi:10.1007/s10482-018-1040-x 29460206 PMC5945754

[54] Nishimura S, Manabe I, Nagasaki M, Eto K, Yamashita H, Ohsugi M, Otsu M, Hara K, Ueki K, Sugiura S, Yoshimura K, Kadowaki T, Nagai R. 2009. CD8^+^ effector T cells contribute to macrophage recruitment and adipose tissue inflammation in obesity. Nat Med 15:914–920. doi:10.1038/nm.1964 19633658

[55] Sun Q, Cai D, Liu D, Zhao X, Li R, Xu W, Xie B, Gou M, Wei K, Li Y, Huang J, Chi X, Wei P, Hao J, Guo X, Pan B, Fu Y, Ni L, Dong C. 2023. BCL6 promotes a stem-like CD8^+^ T cell program in cancer via antagonizing BLIMP1. Sci Immunol 8:eadh1306. doi:10.1126/sciimmunol.adh1306 37862431

[56] Paz Del Socorro T, Oka K, Boulard O, Takahashi M, Poulin LF, Hayashi A, Chamaillard M. 2024. The biotherapeutic Clostridium butyricum MIYAIRI 588 strain potentiates enterotropism of Rorγt^+^Treg and PD-1 blockade efficacy. Gut Microbes 16:2315631. doi:10.1080/19490976.2024.2315631 38385162 PMC10885180

[57] Zhu Z, Huang J, Zhang Y, Hou W, Chen F, Mo Y-Y, Zhang Z. 2024. Landscape of tumoral ecosystem for enhanced anti-PD-1 immunotherapy by gut Akkermansia muciniphila. Cell Rep 43:114306. doi:10.1016/j.celrep.2024.114306 38819989

[58] Reina-Campos M, Scharping NE, Goldrath AW. 2021. CD8^+^ T cell metabolism in infection and cancer. Nat Rev Immunol 21:718–738. doi:10.1038/s41577-021-00537-8 33981085 PMC8806153

[59] Cassetta L, Pollard JW. 2018. Targeting macrophages: therapeutic approaches in cancer. Nat Rev Drug Discov 17:887–904. doi:10.1038/nrd.2018.169 30361552

[60] Bosch FX, Ribes J, Díaz M, Cléries R. 2004. Primary liver cancer: worldwide incidence and trends. Gastroenterology 127:S5–S16. doi:10.1053/j.gastro.2004.09.011 15508102

[61] Rahbari NN, Mehrabi A, Mollberg NM, Müller SA, Koch M, Büchler MW, Weitz J. 2011. Hepatocellular carcinoma: current management and perspectives for the future. Ann Surg 253:453–469. doi:10.1097/SLA.0b013e31820d944f 21263310

[62] Chang C-W, Lee H-C, Li L-H, Chiang Chiau J-S, Wang T-E, Chuang W-H, Chen M-J, Wang H-Y, Shih S-C, Liu C-Y, Tsai T-H, Chen Y-J. 2020. Fecal microbiota transplantation prevents intestinal injury, upregulation of toll-like receptors, and 5-fluorouracil/oxaliplatin-induced toxicity in colorectal cancer. Int J Mol Sci 21:386. doi:10.3390/ijms21020386 31936237 PMC7013718

[63] Routy B, Lenehan JG, Miller WH Jr, Jamal R, Messaoudene M, Daisley BA, Hes C, Al KF, Martinez-Gili L, Punčochář M, et al.. 2023. Fecal microbiota transplantation plus anti-PD-1 immunotherapy in advanced melanoma: a phase I trial. Nat Med 29:2121–2132. doi:10.1038/s41591-023-02453-x 37414899

[64] Kang X, Ng S-K, Liu C, Lin Y, Zhou Y, Kwong TNY, Ni Y, Lam TYT, Wu WKK, Wei H, Sung JJY, Yu J, Wong SH. 2023. Altered gut microbiota of obesity subjects promotes colorectal carcinogenesis in mice. EBioMedicine 93:104670. doi:10.1016/j.ebiom.2023.104670 37343363 PMC10314234

[65] Nguyen N-P, Warnow T, Pop M, White B. 2016. A perspective on 16S rRNA operational taxonomic unit clustering using sequence similarity. NPJ Biofilms Microbiomes 2:16004. doi:10.1038/npjbiofilms.2016.4 28721243 PMC5515256

