# Supplementary material for: The combination of Clostridium butyricum and Akkermansia muciniphila mitigates DSS-induced colitis and attenuates colitis-associated tumorigenesis by modulating gut microbiota and reducing CD8+ T cells in mice
Source: mSystems. 2025 Jan 22;10(2):e01567-24. doi: 10.1128/msystems.01567-24 (PMC11834468; doi:10.1128/msystems.01567-24)
Supplement: Supplemental material — Supplemental tables and figures. [file msystems.01567-24-s0001.docx]

**Supplementary Tables:**

## **Table S1：**

**Supplementary Table 1: DAI Score**

| Body weight loss | Stool consistency | Bleeding | Score |
| --- | --- | --- | --- |
| none | none | none | 0 |
| 0-5% | / | trace | 1 |
| 5-10% | loose | mild hemoccult | 2 |
| 10-20% | / | obvious hemoccult | 3 |
| >20% | diarrhea | gross bleeding | 4 |

Note: Different scores indicate the degree of inflammation, and a higher score indicates severe inflammation.

## **Table S2**

**Supplementary Table 2: Histopathological inflammation scores**

| Epithelial surface change | Infiltration of immunocytes | Ulcer | Lympho-  glandula | Score |
| --- | --- | --- | --- | --- |
| no change | no | 0 | 0 | 0 |
| goblet cells are missing | perpetual crypt infiltration | 1 | 1 | 1 |
| goblet cells are largely absent | mucosal muscular infiltration | 2 | 2 | 2 |
| crypt loss | The muscular mucosa is generally infiltrated, and the mucosa is thickened | 3 | 3 | 3 |
| extensive loss of crypts or polypoid regrowth | Submucosal infiltration | >3 | >3 | 4 |

## **Table S3：**

**Supplementary Table 3: Scale for Degree of Dysplasia***

| Degree of dysplasia | Score |
| --- | --- |
| negative of dysplasia | 0 |
| low-grade dysplasia | 1 |
| high-grade dysplasia | 2 |

*Dysplasia was defined by the presence of hyperchromasia, nuclear pleomorphism, increased nuclear-to-cytoplasmic ratios, and atypical mitotic figures.

## **Table S4：**

**Supplementary Table 4: Antibodies**

| Antibodies | Use | Source | Cat# |
| --- | --- | --- | --- |
| CD45-APC-Cy7 | Flow Cytometry | BD Biosciences | 557659 |
| CD3e-FITC | Flow Cytometry | BD Biosciences | 553061 |
| CD4-APC/BV605 | Flow Cytometry | BD Biosciences | 553051/ 563151 |
| CD8a-PerCP-CY5.5 | Flow Cytometry | BD Biosciences | 551162 |
| CD25-BV650 | Flow Cytometry | BD Biosciences | 552880 |
| CD11b-FITC | Flow Cytometry | BD Biosciences | 553310 |
| F4/80-APC | Flow Cytometry | BD Biosciences | 566787 |
| CD86-PE | Flow Cytometry | BD Biosciences | 553692 |
| Occludin | IHC | Affinity | DF7504 |
| ZO-1 | IHC | Proteintech Group Inc, China | 21773-1-AP |
| Caspase 3 | IHC | Proteintech Group Inc, China | 66470-2-Ig |
| CD4 | IHC | Cell Signaling Technology | 25229T |
| CD8 | IHC | Cell Signaling Technology | 98941T |
| F4/80 | IHC | Cell Signaling Technology | 70076T |
| Ki67 | IHC | Cell Signaling Technology | 12202T |

## **Table S5：**

**Supplementary Table 5: Survival probability of colitis-associated CRC mice in 16th week.**

| Group(16 W) | Untreat | NS | aPD-L1 | Combo | Combo+aPD-L1 |
| --- | --- | --- | --- | --- | --- |
| Probability of Survival | 100% | 100% | 100% | 100% | 100% |

# **Figure S1**


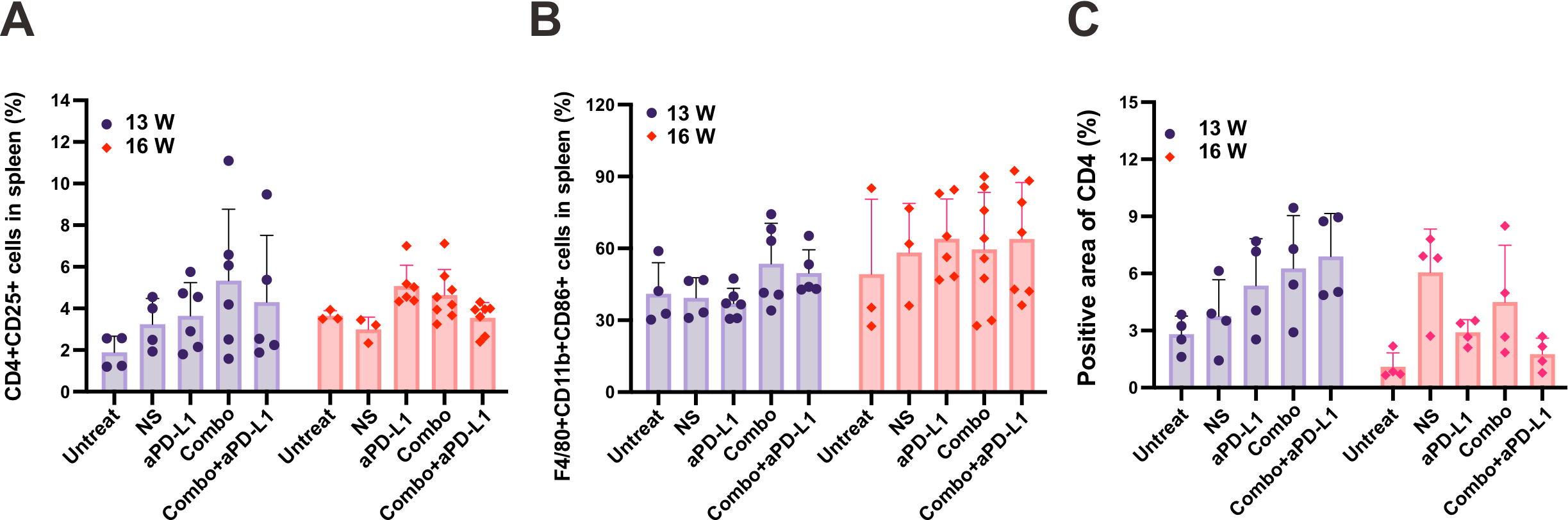


# **Figure S2**


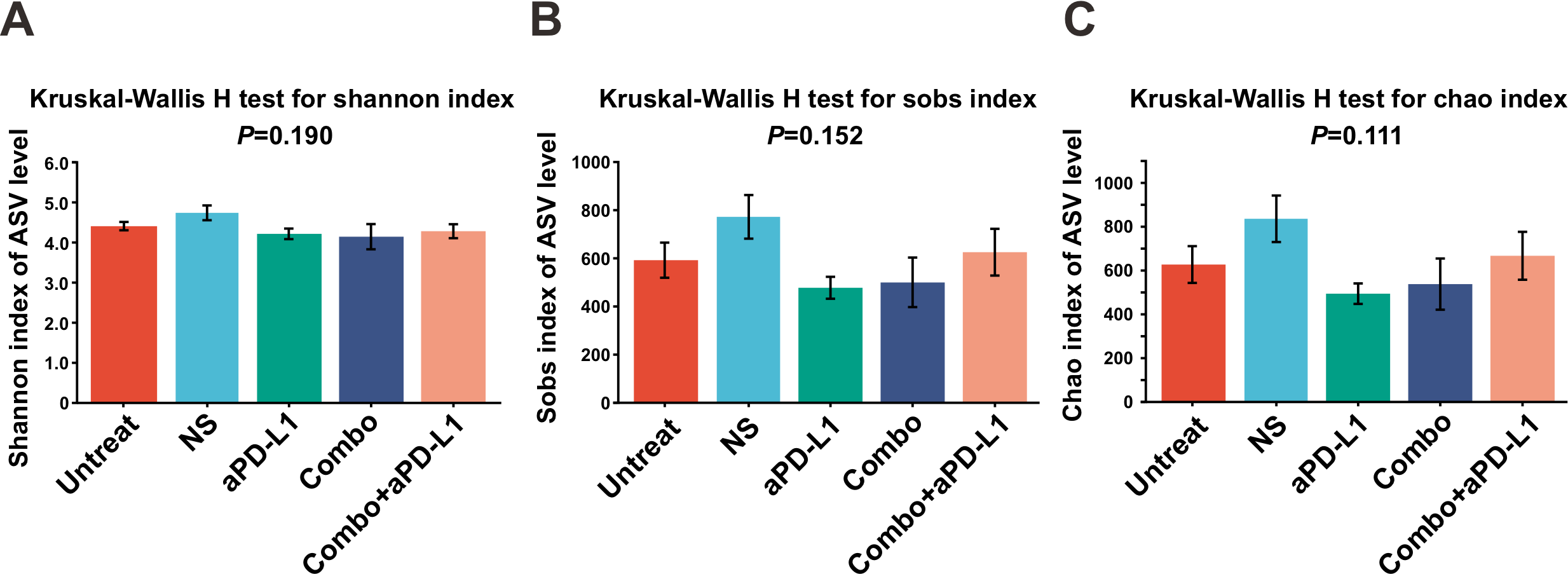


# **Figure S3**


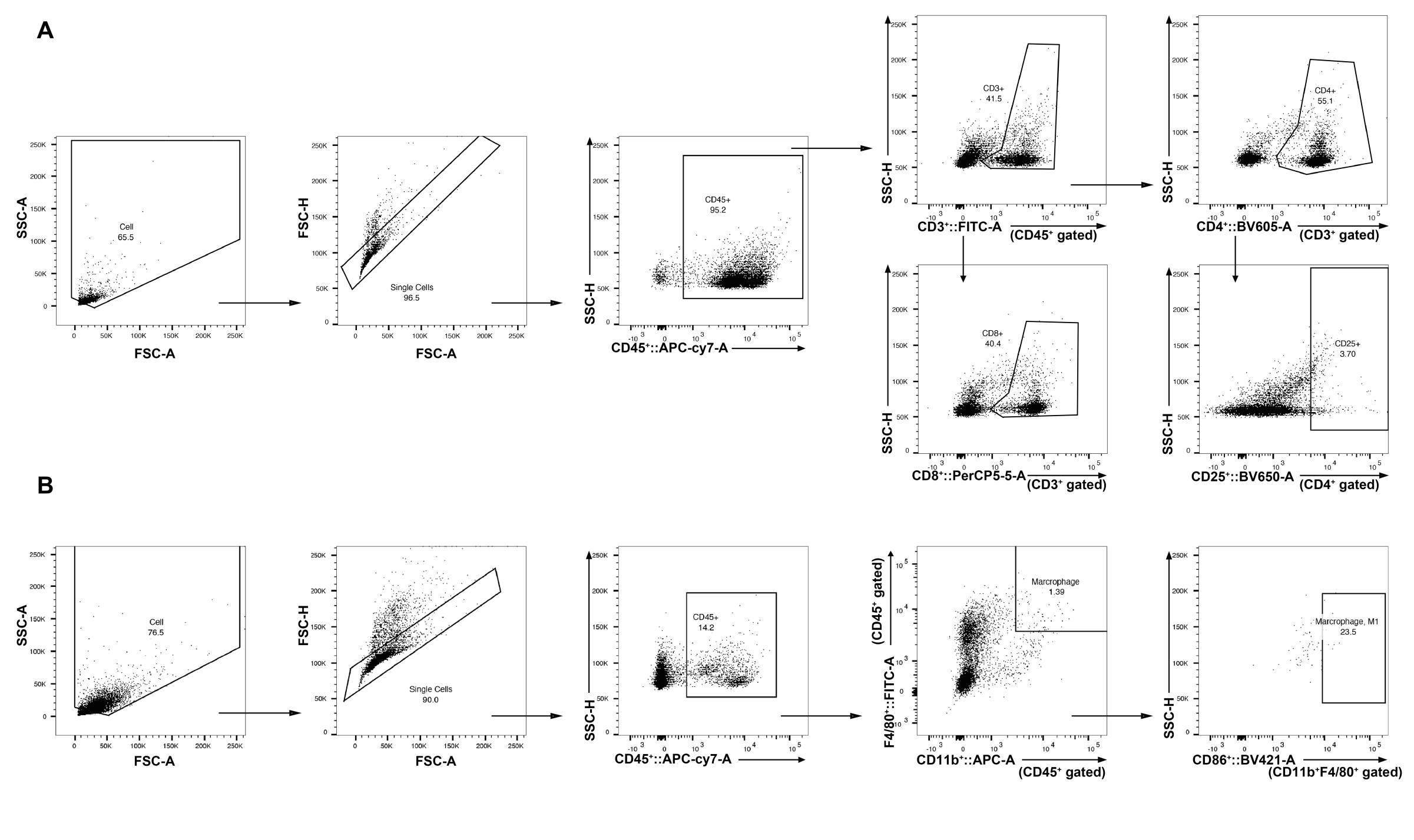


# **Supplementary Figures legends:**

# **Figure S1 Immune-related cells in the spleen or colon of colitis-associated CRC mice were evaluated by FCM and IHC.**

(A) Treg (CD4^+^CD25^+^ T) cells and (B) M1-type Macrophages (CD11b^+^F4/80^+^CD86^+^) in the spleen were quantified by FCM. (C) The IHC-positive staining area of CD4^+^ T in the colon. Data are expressed as mean ±SD. One-way ANOVA was used to determine statistical significance, and then Tukey's test was performed. *P<0.05, **P<0.01, ***P<0.001 or #P<0.05, ##P<0.01, ###P<0.001, compared with NS group.

# **Figure S2 α-diversity index of colitis-associated CRC mice.**

(A) The Shannon index of ASV level. (B) Sobs index of ASV level of mice in each group were compared in the 13th week. (C) Chao index of ASV level of mice in each group were compared in the 13th week.

# **Figure S3 FCM gating strategies.**

(A) Gating strategy for the analysis of the percentage of CD4^+^ T and CD8^+^ T cells in CD3^+^ T cells and CD25^+^ cells in CD4^+^ T cells. (B) Gating strategy for the analysis of the percentage of Macrophage (CD11b^+^F4/80^+^) cells in the spleen, M1 cells (CD86^+^) in macrophage (CD11b^+^F4/80^+^) cells.
